# Cacao pod transcriptome profiling of seven genotypes identifies features associated with post-penetration resistance to *Phytophthora palmivora*

**DOI:** 10.1038/s41598-024-54355-8

**Published:** 2024-02-20

**Authors:** Indrani K. Baruah, Jonathan Shao, Shahin S. Ali, Martha E. Schmidt, Lyndel W. Meinhardt, Bryan A. Bailey, Stephen P. Cohen

**Affiliations:** 1 Sustainable Perennial Crops Laboratory, U.S. Department of Agriculture-Agricultural Research Service, Beltsville, MD 20705 USA; 2 Statistics and Bioinformatics Group-Northeast Area, U.S. Department of Agriculture-Agricultural Research Service, Beltsville, MD 20705 USA; 3 ATCC (American Type Culture Collection), Gaithersburg, MD 20877 USA

**Keywords:** RNA sequencing, Biotic, Plant immunity, Gene regulation, Gene expression profiling, Agricultural genetics

## Abstract

The oomycete *Phytophthora palmivora* infects the fruit of cacao trees (*Theobroma cacao*) causing black pod rot and reducing yields. Cacao genotypes vary in their resistance levels to *P. palmivora*, yet our understanding of how cacao fruit respond to the pathogen at the molecular level during disease establishment is limited. To address this issue, disease development and RNA-Seq studies were conducted on pods of seven cacao genotypes (ICS1, WFT, Gu133, Spa9, CCN51, Sca6 and Pound7) to better understand their reactions to the post-penetration stage of *P. palmivora* infection. The pod tissue-*P. palmivora* pathogen assay resulted in the genotypes being classified as susceptible (ICS1, WFT, Gu133 and Spa9) or resistant (CCN51, Sca6 and Pound7). The number of differentially expressed genes (DEGs) ranged from 1625 to 6957 depending on genotype. A custom gene correlation approach identified 34 correlation groups. De novo motif analysis was conducted on upstream promoter sequences of differentially expressed genes, identifying 76 novel motifs, 31 of which were over-represented in the upstream sequences of correlation groups and associated with gene ontology terms related to oxidative stress response, defense against fungal pathogens, general metabolism and cell function. Genes in one correlation group (Group 6) were strongly induced in all genotypes and enriched in genes annotated with defense-responsive terms. Expression pattern profiling revealed that genes in Group 6 were induced to higher levels in the resistant genotypes. An additional analysis allowed the identification of 17 candidate *cis*-regulatory modules likely to be involved in cacao defense against *P. palmivora*. This study is a comprehensive exploration of the cacao pod transcriptional response to *P. palmivora* spread after infection. We identified cacao genes, promoter motifs, and promoter motif combinations associated with post-penetration resistance to *P. palmivora* in cacao pods and provide this information as a resource to support future and ongoing efforts to breed *P. palmivora*-resistant cacao.

## Introduction

Cacao (*Theobroma cacao* L.) farms are affected by black pod rot (BPR), a devastating disease caused by multiple *Phytophthora* species, among which *Phytophthora palmivora* (Ppal) is the most widespread^1^. Ppal attacks all cacao parts, including pods, leaves, stems and branches, but is most damaging on pods^2^. Cacao pods are harvested at maturity for their seeds, commonly known as cocoa beans, which are processed to make chocolate. Estimates of cacao fruit yield losses due to *Phytopthora* diseases are approximately 30%, or 3.8 billion USD in value^3^.

Early studies into cacao resistance to BPR explored the differential responses of cacao in response to different inoculations and inoculum sources^4^. This early work led to the discovery that cacao polyphenol oxidase activity was an important component of the Ppal resistance response^5^. Inoculation methods were later developed to distinguish the two distinct stages of infection: the penetration stage and the post-penetration stage^6^. In resistance during the penetration stage, morphological characteristics and other resistance factors restrict the entry and establishment of the pathogen, reducing the frequency of lesions. During post-penetration resistance, also called spread resistance, the spread of the pathogen is restricted via mechanisms unique from penetration resistance. These two modes of resistance are independently controlled and there are likely multiple mechanisms of resistance^7–9^. Pod morphological characteristics like stomatal frequency and pore length, surface wax, hardness, thickness have been characterized to understand how resistance mechanisms differ by cacao genotypes^7^. Morphological characteristics such as stomatal frequency and pore length were correlated with lesion formation but not expansion, indicating that penetration resistance, but not post-penetration resistance, is influenced by pod physical characteristics. However further investigations are required to identify both the genetic basis and additional factors responsible for post-penetration resistance.

Measurement of resistance to BPR and identification of resistant cacao genotypes is determined through disease incidence in the field or by measuring lesion size following artificial inoculation of twigs, leaves or attached or detached pods^10–17^. Cacao histological traits including the number of cells in epicarp and number of vascular bundles are reliable indicators for resistance screening^18^. BPR resistance is inherited in a quantitative manner, i.e. as quantitative trait loci (QTLs)^19^. In a meta-analysis study, 13 consensus QTLs were identified from a total of 65 *Phytophthora-*resistance QTLs spread over all of cacao’s 10 chromosomes, supporting the hypothesis that BPR resistance is quantitative^20^. Resistance to one Phytophthora species contributes to resistance to all Phytophthora species and cacao genotypes show similar resistance or tolerance responses irrespective of the infecting species of *Phytophthora*^13,14,21–23^. However, the molecular mechanisms of these QTLs remain to be characterized, because disease resistance QTLs are only defined in terms of genome position and effect of resistance.

Previous studies have established a starting point to identify suites of *Phytophthora-*responsive cacao genes for future studies to observe the diverse reactions of cacao genotypes showing resistance or tolerance to infection by *Phytophthora* spp.^21,24–29^. RNA-Seq experiments on pod pieces infected by Ppal and *P. megakarya* indicated the importance of genes in several metabolic and response pathways, including phenylpropanoid biosynthesis, ethylene and jasmonic acid biosynthesis and action, plant defense signal transduction, as well as a subset of genes encoding pathogenesis-related (PR)-proteins^21^. Transcriptomic analysis of the cacao response to Ppal in the resistant genotype Sca6 and susceptible genotype NA32 revealed a transcriptomic response involving PR genes, pattern recognition receptors, and resistance genes in the resistant genotype^29^. Although thousands of genes involved in cacao biotic response have been identified, studies utilizing both resistant and susceptible cacao genotypes are required to clarify key molecular players that mediate resistance or tolerance so that molecular breeding programs can benefit and improve cacao resistance to Ppal.

While identifying differentially regulated genes is of interest to understanding how plants respond to disease stress, there is also a need to understand how non-coding regions of plant genomes act to direct gene expression^30^. Understanding how plant promoters are organized and how regulatory DNA elements interact will allow rapid advancement of crops via both conventional breeding and the design and deployment of synthetic promoters^31,32^. Identification and characterization of promoter elements unique to cacao is limited. Transcriptional screening of ESTs was used to identify a single motif (ATTSCAMYATCWGC) that was a likely candidate for activation of protease inhibitors in cacao during response to *P. megakarya* infection^33^. Five novel motifs were found in the cacao family of GASA genes, genes involved in a myriad of plant processes including both standard growth and development and also resistance to abiotic and biotic stresses^34^. Another study described regulatory motifs found upstream of magnesium transporter genes in cacao and two closely-related species which were primarily classified as low temperature, anaerobic stress, and biotic stress response motifs^35^. However, to date there has been no in-depth identification of cacao *P. palmivora*-responsive *cis*-regulatory elements.

The current study used a wounded, detached pod assay to bypass the penetration stage providing and assess post-penetration resistance^36^ in pods of seven cacao genotypes. In this study, we characterized the cacao pod transcriptome response to Ppal post-penetration spread in the following genotypes: Imperial College Selection 1 (ICS1), White Flower Tree (WFT, a white-flowered and -seeded Brazilian Amelonado maintained in the SPCL collection), Gu133, Spa9, Coleccion Castro Naranjal 51 (CCN51), Scavina 6 (Sca6) and Pound7. In addition to identifying suites of differentially regulated genes, we conducted de novo motif discovery analysis on the upstream regions of induced genes to identify novel regulatory elements. Our objective was to utilize diverse cacao genotypes to identify genes and regulatory DNA elements responsible for the activation of the cacao pod defense transcriptome. This work is an important resource for the identification of candidate genes and promoter elements for use in molecular assisted breeding programs to develop cacao resistance to Ppal.

## Results

### Levels of resistance to *P. palmivora* in pods varied by genotype

**Figure 1:**
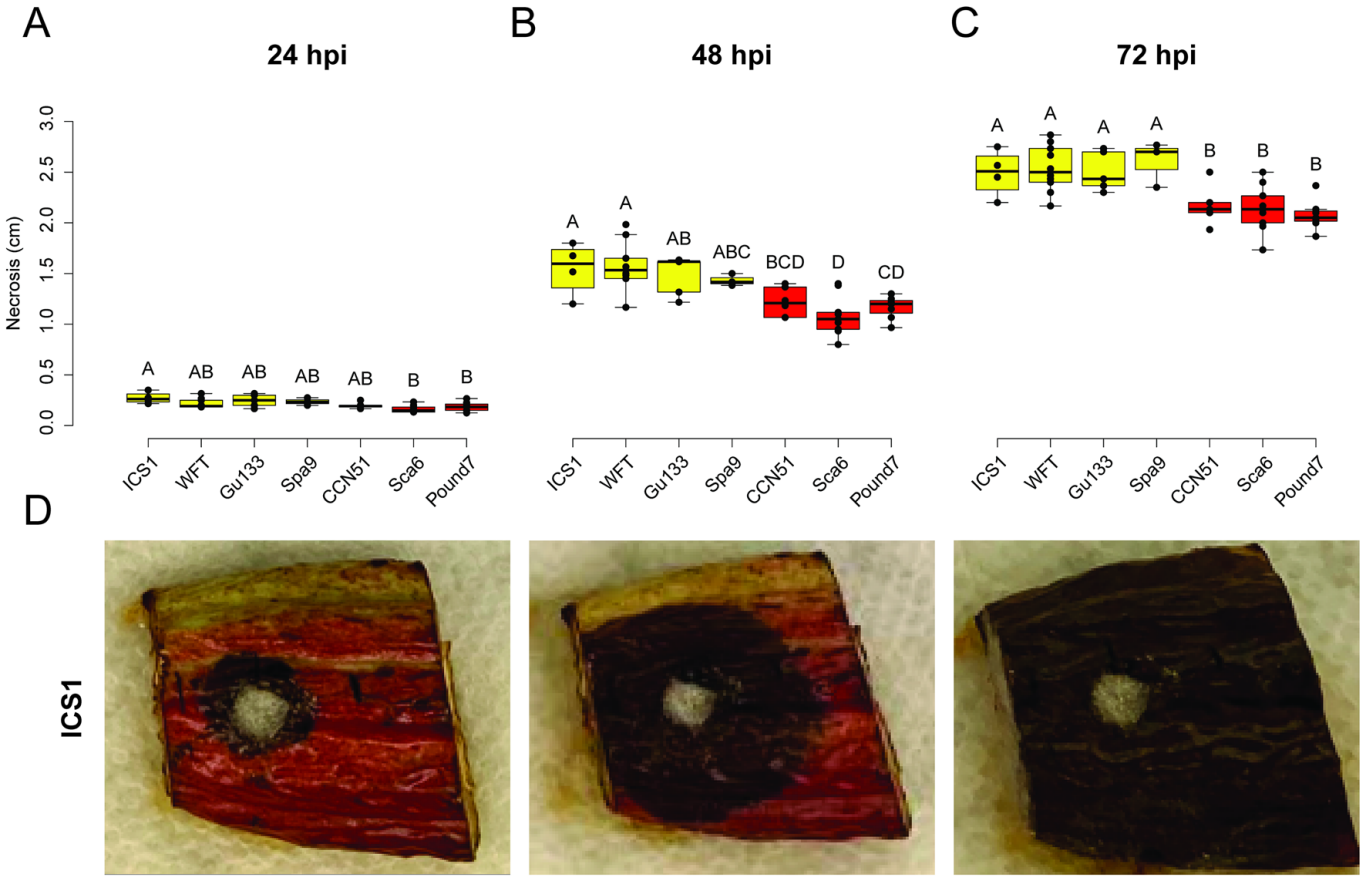
Cacao response to Ppal in seven genotypes. Necrosis quantified at (**A**) 24 hpi, (**B**) 48 hpi, and (**C**) 72 hpi; statistical groups were determined with the Student–Newman–Keuls test. (**D**) Representative necrosis symptoms of pod pieces from genotype ICS1 at 24, 48, and 72 hpi.

The seven cacao genotypes studied—ICS1, WFT, Gu133, Spa9, CCN51, Sca6 and Pound7 varied in their reactions to *P. palmivora* (Ppal) pod infection. The average necrotic lesion size from the point of plug inoculation per pod piece was observed and measured at 3 different time points—24 hpi, 48hpi and 72 hpi (Supplementary Fig. S1). At 24 hpi, there were three groups based on lesion lengths: susceptible (higher necrosis) in ICS1, resistant (lower necrosis) in Sca6 and Pound7, and intermediate lesion lengths in WFT, Gu133, Spa9, and CCN51 (Fig. 1A). By 48 hpi, the separation of susceptible (ICS1 and WFT) and resistant (Sca6 and Pound7) was more evident, with only three genotypes (Gu133, Spa9, and CCN51) showing intermediate lesion lengths (Fig. 1B). By 72 hpi, only two distinct phenotypes were evident: susceptible (in genotypes ICS1, WFT, Gu133, and Spa9), and resistant (in genotypes CCN51, Sca6, and Pound7) (Fig. 1C). Pod samples with representative symptoms are shown for ICS1 in Fig. 1D and for all genotypes in Supplementary Fig. S1.

### The cacao pod Ppal infection transcriptome

We generated 42 RNA-Seq libraries from RNA extracted from pods of seven genotypes at 48 h after treatment (Ppal colonized agar plugs) and control (uninoculated agar plugs), three replications per treatment and control per genotype. The libraries ranged in size from 40.7 M to 47.9 M raw reads (Supplementary Table S1). Reads were mapped to the Matina 1–6 cacao genome V2.0 (available at Phytozome by the HudsonAlpha Institute for Biotechnology in collaboration with MARS, Incorporated), which consists of 27,379 putative feature-coding genes. The number of reads per sample mapped to features ranged from 14.0 M to 26.5 M (Supplementary Table S1).

**Figure 2:**
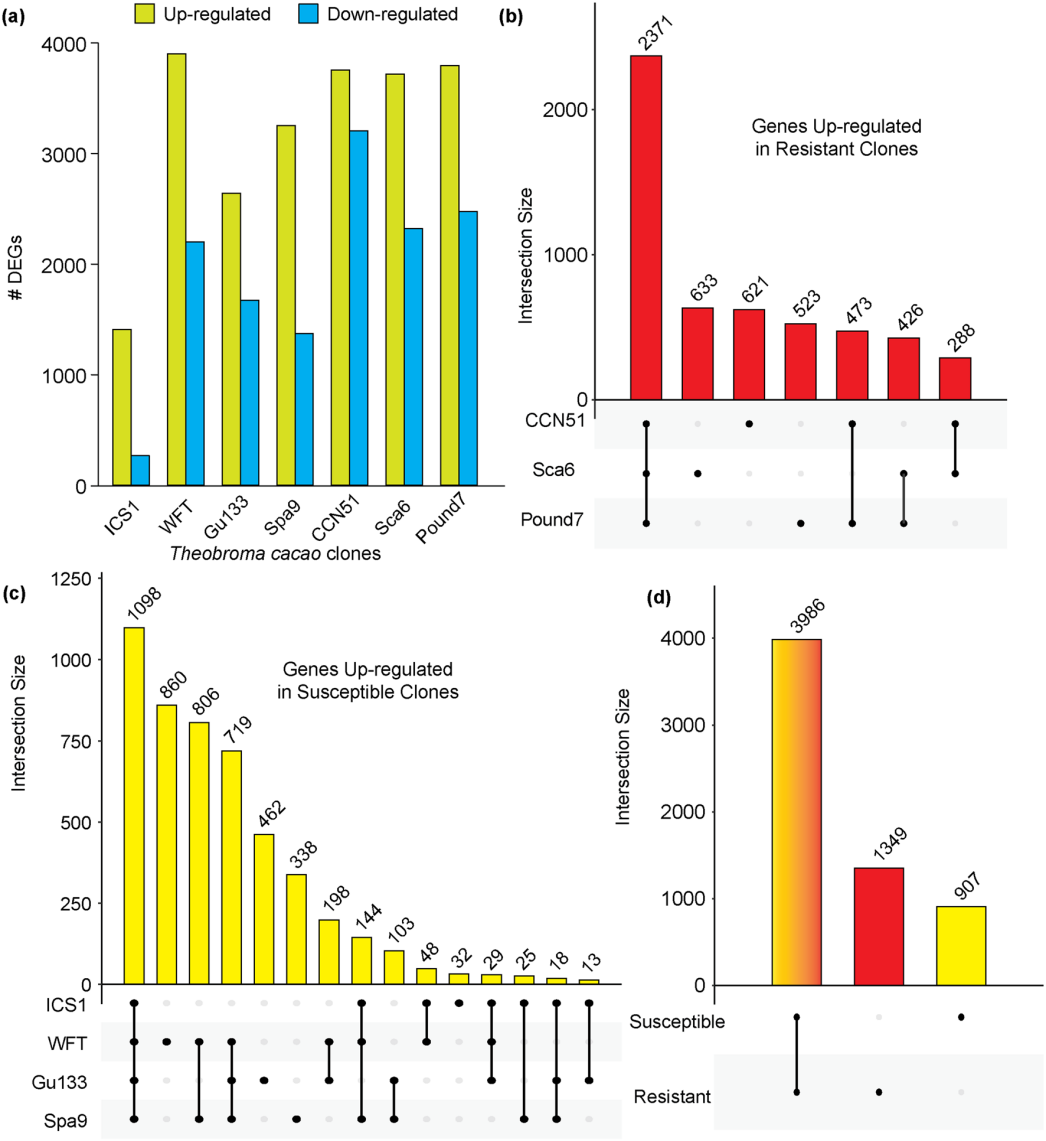
Differential gene expression analysis reveals unique transcriptome responses of seven genotypes after inoculation with Ppal. (**a**) DGE analysis for all seven genotypes. (**b**) Overlap in numbers of up-regulated genes in resistant genotypes. (**c**) Overlap in numbers of up-regulated genes in susceptible genotypes. (**d**) Overlap in numbers of genes up-regulated in at least one susceptible and/or at least one resistant genotype.

Differential gene expression analysis was used to characterize similar and unique gene expression patterns in pods of all seven cacao genotypes selected for this study in response to Ppal. For each cacao genotype, the transcriptome profiles of Ppal-treated pods were compared to the transcriptome profiles of control pods of the same genotype to determine genes differentially expressed in response to Ppal infection. The number of differentially expressed genes (DEGs) in susceptible genotypes were 1625 in ICS1, 6104 in WFT, 4314 in Gu133 and 4623 in Spa9 (Fig. 2a). In resistant genotypes, the number of DEGs were 6957 in CCN51, 6041 in Sca6 and 6267 in Pound7 (Fig. 2a). The numbers of genes uniquely up-regulated by a single resistant genotype were 633 (Sca6), 621 (CCN51), and 523 (Pound7) (Fig. 2b). There were totals of 1098 and 44 genes up- and down-regulated, respectively in all four susceptible genotypes (Fig. 2c; Supplementary Fig. S2b). The numbers of genes uniquely up-regulated by a single susceptible genotype were 32 (ICS1), 860 (WFT), 462 (Gu133) and 338 (Spa9) (Fig. 2c). There were 3986 total genes up-regulated in at least one resistant and at least one susceptible genotype, 1349 in at least one resistant genotype but no susceptible genotypes, and 907 in at least one susceptible genotype but no resistant genotypes (Fig. 2d). There were 2435 total genes down-regulated in at least one resistant and at least one susceptible genotype, 2160 in at least one resistant genotype but no susceptible genotypes, and 1500 in at least one susceptible genotype but no resistant genotypes (Supplementary Fig. 2c). The detailed breakdown of numbers of up- and down-regulated genes in resistant genotypes are shown in Supplementary Fig. S2d and in susceptible genotypes are shown in Supplementary Fig. S2e.

### Gene correlation analysis

To find groups of similarly regulated genes, a custom gene correlation approach was used with normalized RNA-seq gene expression data. The correlation analysis resulted in 34 correlation groups. The number of genes in all correlation groups ranged from 40 (group 32) to 4533 (group 1) (Supplementary Fig. S3). Correlation groups assigned to all genes are shown in Supplementary Data 1. The mean log_2_ fold changes of genes in groups 6, 10, 11, 19, 22, 27 and 34 were positive in all or most genotypes, indicating that genes in these groups are generally up-regulated. The mean log_2_ fold changes of genes in group 1 were negative, indicating that genes in this group are generally down-regulated (Supplementary Fig. S3). These 34 groups were tested for over-representation of specific gene families within groups. Genes predicted to encode leucine-rich repeats (LRRs) were over-represented in groups 2, 4, 12, 13, 14, 16, 17, 19, 20 and 24 while PR10s, Germin-like proteins (GLPs) and WRKYS were over-represented only in Group 6 (Supplementary Fig. S3). Genes encoding MAPKs were over-represented in both groups 6 and 10 while genes encoding ERFs were over-represented in groups 10 and 11. Genes encoding cytochrome P450 proteins (CYPs) were over-represented in group 28, Glutathione-S-transferases (GSTs) in groups 22 and 34 and Ubiquitin-related proteins in groups 4, 6, 7, 10 and 30 (Supplementary Fig. S3). LRRs, PR10s, GLPs, CYPs, and ERFs were under-represented in group 1, indicating that genes in these families are generally not down-regulated. The number of genes differentially regulated within these families did not vary much per genotype (Supplementary Table S2), however there were some notable differences, including higher overall numbers of LRRs and ubiquitin-related genes up-regulated in resistant genotypes.

### Promoter motif discovery

De novo motif discovery analysis was conducted on the upstream promoter sequences of up- and down-regulated genes from each of the seven cacao genotypes; upstream regions were the genomic regions upstream of the putative transcription start site (TSS) from the cacao Matina 1–6 reference genome^37^. There were 76 motifs discovered from all DEG sets (Supplementary Table S3). Enrichment analysis revealed that 42 of the motifs were over-represented in the upstream sequences of genes in the 34 correlation groups (Supplementary Table S3). All motifs were tested for association with gene ontology (GO) terms in plants, and 31 of the 42 overrepresented motifs were associated with GO terms (Table 1). Motifs over-represented in the upstream sequences of correlation groups 10, 23, and 6 were associated with GO terms indicative of oxidative stress response, including ‘mitochondrion’, ‘mitochondrial transport’, and ‘chloroplast’ (Supplementary Fig. S4). Motifs overrepresented in group 6 were also associated with GO terms indicative of general stress response (‘response to heat’, ‘response to wounding’), defense against fungal pathogens (‘response to chitin’, ‘defense response’, ‘polygalacturonase activity’), protein and RNA synthesis and turnover (‘translation’, ‘transcription’, ‘DNA-directed RNA polymerase activity’, ‘CUL4 RING ubiquitin ligase’) and others (Supplementary Fig. S4). Occurrences of these 31 de novo motifs and 229 motifs from the PLACE database were quantified for all upstream sequences from cacao (sheets 1 and 2 from Supplementary Data 1).

**Table 1:** Motifs and associated gene ontology terms discovered from differentially expressed gene sets.

Motif	Correlation group*	GO term associations**
GACTTTGTCAA	6	DR, KA
CTTCTAGAA	6	RH
TGGTCAAAD	6	DR, KA
GAAAAGTCAAAA	6	CaB, KA
CAAGGAAA	6	TR, PK
GGTCAAAN	6	KA
GAAAAGTC	6	KA
ACACGYTW	(1), 6	RD, RW
VAAAGTCAA	6	DR, ES, KA
ADMCGCGKHT	6, 10, 23	C, M
AGCCGCCA	6	BB, C4, CS, HA, LR, MIM, NB, Nu, RB, RP, SR, Ts, Tx
AAAATAATACT	6	ES, RT, TF
DMCGCGKH	(1), 6, 10, 23	C, M
AGAAAAGTCTM	6	M
MGCCGCCA	6	AH, C4, CE, CS, LR, MIM, NB, Nu, OR, PT, RB, RP, SR, TM, Ts, TsF, Tx
AASCGCGTKGRV	6	C, M
CGCCGCCN	6	AH, C4, CE, CS, LR, MIM, MT, NB, Nu, RB, RP, SR, Ts, TsF, Tx
CTATAAATACCCM	6	CaM, CL, CM, ER, ES, LB, LT, OD, OT, PA, PI, PS, PX, RA, TF, WC
TTGGTCAAAMHVR	6	CR
WGGTCAAA	6	KA
ACGCGGYK	6, 10	C, M, MT, NB, SR, Ts
AGTCAAMG	(1), 6	DR, KA
TGGTCAAMMD	6	KA
AAGTCAAAA	6	CR, DR, ES, KA
TCTAGAAGG	6	M, RH, Ts
AGTCTTTG	6	DR
ACCGACCD	6	C, M
GAARYTTCCACG	6	C, RH
CTTTGACTW	6	DR
MGCCGCCR	6	AH, C4, CE, CS, ED, LR, MIM, NB, Nu, RB, RP, SR, Ts, TsF, Tx
CTATAAATACCM	6	CaM, CL, CM, ES, LB, LT, PA, RA, TF

Discovered motifs were tested for overrepresentation in correlation groups via Fisher’s exact test, and for associations with GO terms with GOMo.

*Motifs are considered overrepresented or underrepresented (parentheses) in correlation groups following two-sided Fisher’s exact test (p ≤ 0.05); **Associated GO term abbreviations: AH (ATP-dependent helicase activity), BB (biotin biosynthetic process), C (chloroplast), C4 (CUL4 RING ubiquitin ligase complex), CaB (carbohydrate binding), CaM (carbohydrate metabolic process), CE (chloroplast envelope), CL (plant-type cell wall loosening), CM (plant-type cell wall modification during multidimensional cell growth), CR (response to chitin), CS (chloroplast stroma), DR (defense response), ED (embryonic development ending in seed), ER (extracellular region), ES (endomembrane system), HA (helicase activity), KA (kinase activity), LB (lipid binding), LR (cytosolic large ribosomal subunit), LT (lipid transport), M (mitochondrion), MIM (mitochondrial inner membrane), MT (mitochondrial transport), NB (nucleotide binding), Nu (nucleolus), OD (multicellular organismal development), OR (oxidoreductase activity), OT (oligopeptide transport), PA (polygalacturonase activity), PI (pectinesterase inhibitor activity), PS (cellular response to phosphate starvation), PT (protein transporter activity), PX (peroxidase activity), RA (response to auxin stimulus), RB (RNA binding), RD (response to water deprivation), RH (response to heat), RP (DNA-directed RNA polymerase activity) RT (regulation of transcription), RW (response to wounding), SR (structural constituent of ribosome), TF (transcription factor activity), TM (chloroplast thylakoid membrane), TR (transmembrane receptor protein tyrosine kinase signaling pathway), Ts (translation), TsF (translation initiation factor activity), Tx (transcription), WC (water channel activity).

### Expression pattern profiling for all genes

**Figure 3:**
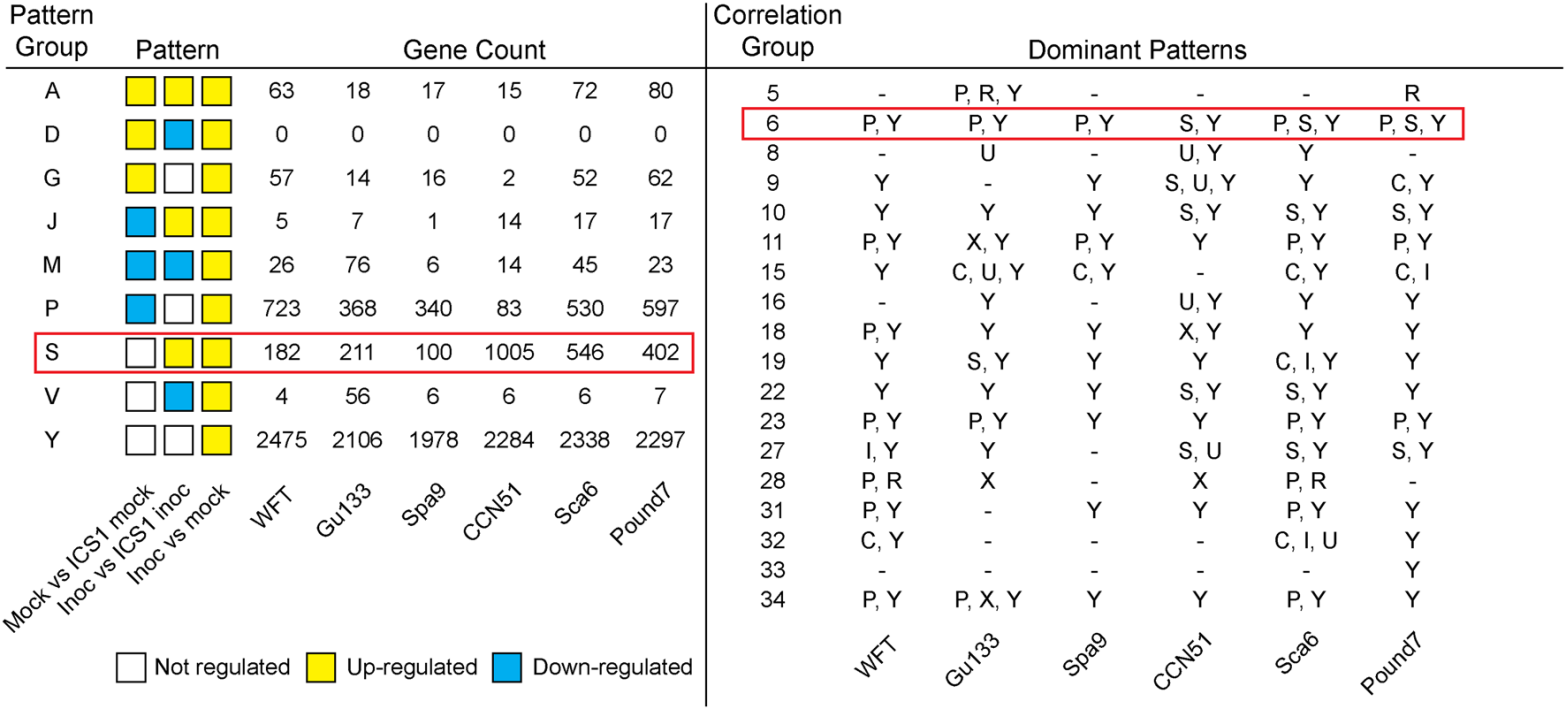
Selected gene expression pattern profiling identifies regulatory trends within correlation groups. (left panel) Gene expression patterns were profiled for all genes in genotypes to test expression of genotype mock vs. ICS1 mock, genotype inoculated vs. ICS1 inoculated, and genotype inoculated vs. genotype mock. Patterns with up-regulation in the genotype (the third comparison) are shown here while all patterns are shown in Supplementary Fig S5. The red box highlights expression pattern S, which indicated genes up-regulated in both the genotype of interest and ICS1, but up-regulated to a higher level in the genotype of interest. Pattern S shows the most notable difference between susceptible and resistant genotypes. (right panel) The presence of dominant expression patterns was profiled for correlation groups. An expression pattern was considered dominant if either 100 + genes or 10 + % of the total genes in the group showed that pattern. Correlation groups containing patterns S and Y as dominant expression patterns are shown here while all groups are shown in Supplementary Fig. S5. Groups with these patterns were chosen for display because they represent groups of defense response genes. Group 6 is highlighted with a red box because this group shows pattern Y in all varieties, pattern S in all resistant varieties, and this group was chosen for follow-up analyses.

To understand how gene expression differs with or without pathogen infection, three comparisons were made for six of the cacao genotypes (referred to as “genotype” below), excluding ICS1: (1) ICS1 mock (control) vs. "genotype" mock (control), (2) ICS1 inoculated vs. "genotype" inoculated, and (3) "genotype" inoculated vs. "genotype" mock (control) (Fig. 3 and Supplementary Fig. S5). ICS1 was chosen as the baseline for these comparisons because of the low number of genes differentially expressed in the ICS1 transcriptome response compared to the other genotypes (Fig. 2a). Genes were considered up- or down-regulated in each comparison if the log_2_ fold change was positive or negative, respectively, and not-regulated if the FDR-corrected p-value was > 0.05 (Fig. 3). A total of 27 possible patterns of expression, named pattern groups A-Z, were determined for all genes in all genotypes, and the numbers of genes matching each pattern for each genotype were quantified (Supplementary Fig. S5). The distribution of patterns was observed in all 34 correlation groups and dominant patterns (patterns with ≥ 100 genes or ≥ 10% of the total number of genes per group) were characterized for each correlation group for all genotypes (Supplementary Fig. S5). Genes classified as **Pattern S**, genes up-regulated in comparisons 2 and 3, are considered the most likely disease resistance-related genes because there were approximately four times more **Pattern S** genes among the resistant genotypes CCN51 (1005), Sca6 (546) and Pound7 (402) compared to susceptible genotypes WFT (182), Gu133 (211), and Spa9 (100) (Fig. 3, left panel). **Pattern S** was dominant in genes belonging to correlation groups 6, 10, 22 and 27 in resistant genotypes (Fig. 3, right panel). Correlation group 6 was particularly of interest because the average log_2_ fold change was positive and dramatic (Supplementary Fig. S3), and **Pattern S** was dominant in group 6 in resistant genotypes, indicating that genes in group 6 were generally up-regulated and to a higher level in resistant genotypes than in susceptible genotypes. Expression patterns assigned to all genes for all genotypes are in Supplementary Data 1.

### Identification of candidate *cis*-regulatory modules

To identify potential *cis-*regulatory modules (CRMs), i.e. functionally related motifs, two analyses of motif positions relative to the transcription start sites (TSS) were conducted. In the first analysis, motifs were identified that were statistically closer to the TSS in induced genes than they were to the TSS in non-induced genes. There were five motifs from the de novo discovery and 9 known motifs from the PLACE database that were closer to the TSS in induced genes (Table 2, Supplementary Data 2). These motifs were associated with GO terms related to oxidative stress response (such as ‘chloroplast’, ‘damaged DNA repair’, ‘mitochondrion’, ‘mismatch repair’, ‘response to auxin stimulus’), defense against fungal pathogens (‘defense response’, ‘polygalacturonase activity’), general metabolism and cell function (‘kinase activity’, ‘lipid transport’, ‘transcription factor activity’) and others.

**Table 2:** A list of discovered and known (PLACE) motifs that are closer to the TSS in induced genes vs. non-induced genes.

Motif	Name	Associated terms*	Induced genes		Non-induced genes		Mean position	
			Count	Mean position	Count	Mean position	Difference	P-value
AGTCTTTG	De novo	DR	79	− 373	268	− 501	128	3.8 × 10^–4^
ATTCGCGC	PE2FNTRNR1A	C, DD, M, DI, MR	2	− 24	11	− 460	436	9.2 × 10^–4^
CTATAAATAC	TATABOX1	ES, LT, PA, RA, TF	51	− 118	99	− 232	114	1.8 × 10^–3^
GAAAAGTC	De novo	KA	117	− 350	334	− 441	91	2.4 × 10^–3^
RGTGACNNNGC	ARE1	C, M	20	− 292	120	− 455	163	2.6 × 10^–3^
VAAAGTCAA	De novo	DR, ES, KA	233	− 411	774	− 470	59	3.5 × 10^–3^
DMCGCGKH	De novo	C, M	331	− 320	679	− 372	52	4.1 × 10^–3^
TGACGTGG	HEXAT	C	54	− 273	245	− 366	93	4.3 × 10^–3^
GGTCANNNAGTC	ELRENTCHN50	–	5	− 213	19	− 442	229	8.4 × 10^–3^
TCCACGTACT	O2F3BE2S1	–	3	− 120	5	− 527	407	1.3 × 10^–2^
AGTCAAMG	De novo	DR, KA	142	− 393	422	− 450	57	1.7 × 10^–2^
GCGTNNNNNNNACGC	VOZATVPP	M, PI	12	− 173	46	− 287	114	2.1 × 10^–2^
CCACGTCA	UPRMOTIFIAT	C	68	− 267	279	− 329	62	3.8 × 10^–2^
CACGCAAT	CACGCAATGMGH3	–	21	− 462	153	− 577	115	4.8 × 10^–2^

Proximity to TSS of all PLACE and discovered motifs were compared in induced genes vs. non-induced genes via Student’s t-test. Associated GO terms, count and mean position for both induced and non-induced genes, difference in mean positions, and t-test p-value are shown for all motifs with position closer (p < 0.05) to the TSS in induced genes.

*Top five associated GO terms, abbreviations: C (chloroplast), DD (damaged DNA repair), DI (DNA replication initiation), DR (defense response), ES (endomembrane system), KA (kinase activity), LT (lipid transport), M (mitochondrion), MR (mismatch repair), PA (polygalacturonase activity), PI (protein import into nucleus), RA (response to auxin stimulus), TF (transcription factor activity), or a dash for no associated terms.

In the second analysis, motifs were identified that were statistically closer to the TSS in genes induced in resistant genotypes than they were to the TSS in genes not induced in resistant genotypes. There were six motifs from the de novo discovery and 16 motifs from PLACE that were closer to the TSS in resistance-induced genes (Table 3, Supplementary Data 3). Of these motifs closer to TSS in resistance-induced genes, associated GO terms included a more robust response to oxidative stress (‘chloroplast’, ‘chlorophyll binding’, ‘damaged DNA repair’, ‘light-harvesting complex’, ‘mitochondrion’, ‘mismatch repair’, ‘photosynthesis’, ‘response to auxin stimulus’), a more broad response to stress (‘response to cold’, ‘response to stress’), and terms related to defense against fungal pathogens (‘defense response’, ‘polygalacturonase activity’, ‘response to chitin’). Four de novo and five PLACE motifs were identified in both motif position analyses.

**Table 3:** A list of discovered and known (PLACE) motifs that are closer to the TSS in genes induced in resistant genotypes vs. genes not induced in resistant genotypes.

Motif*	Name*	Associated terms**	Induced tolerant		Not induced tolerant		Position	
			Count	Mean position	Count	Mean position	Difference	P-value
**ATTCGCGC**	PE2FNTRNR1A	C, DD, M, DI, MR	2	− 24	11	− 460	436	0.000918
TGACGTGGC	AUXRETGA2GMGH3	CB, LC, P, RS, T	55	− 211	90	− 330	119	0.001417
**GAAAAGTC**	De novo	KA	171	− 370	280	− 447	77	0.004631
**TGACGTGG**	HEXAT	C	96	− 297	203	− 374	77	0.008391
**CTATAAATAC**	TATABOX1	ES, LT, PA, RA, TF	70	− 140	80	− 239	99	0.008874
TCCAACTTGGA	RBENTGA3	–	2	− 181	5	− 636	455	0.009201
**DMCGCGKH**	De novo	C, M	506	− 333	504	− 376	43	0.009954
AAGTCAAAA	De novo	DR, CR, ES, KA	178	− 430	322	− 491	61	0.011468
TCTCTCTCT	CTRMCAMV35S	N, PK, PP, RT, TF	302	− 324	787	− 369	45	0.01268
**VAAAGTCAA**	De novo	DR, ES, KA	372	− 430	635	− 473	43	0.012768
**TCCACGTACT**	O2F3BE2S1	–	3	− 120	5	− 527	407	0.012966
**CCACGTCA**	UPRMOTIFIAT	C	114	− 276	233	− 337	61	0.018758
**AGTCTTTG**	De novo	DR	132	− 432	215	− 496	64	0.025345
CTGAAGAAGAA	TL1ATSAR	C, KA, PM	3	− 106	10	− 418	312	0.035129
TCCATGCAT	SPHCOREZMC1	DR, ES, NR, TF	32	− 424	77	− 536	112	0.035266
ACGTGGCA	LRENPCABE	CB, LC, RC, TL, TM	174	− 288	368	− 333	45	0.041094
AGGAATTCCT	HSELIKENTGLN2	–	3	− 92	15	− 383	291	0.043228

Proximity to TSS of all PLACE and discovered motifs were compared in genes induced in resistant genotypes vs. genes not induced in resistant genotypes via Student’s t-test. Associated GO terms, count and mean position for both induced and non-induced genes, difference in mean positions, and t-test p-value are shown for all motifs with position closer (p < 0.05) to the TSS in induced genes. Bold-highlighted motifs were also significantly closer to the TSS in the previous test (see Table 2).

*Motifs in bold were previously identified in the analysis shown in Table 2.

**Figure 4:**
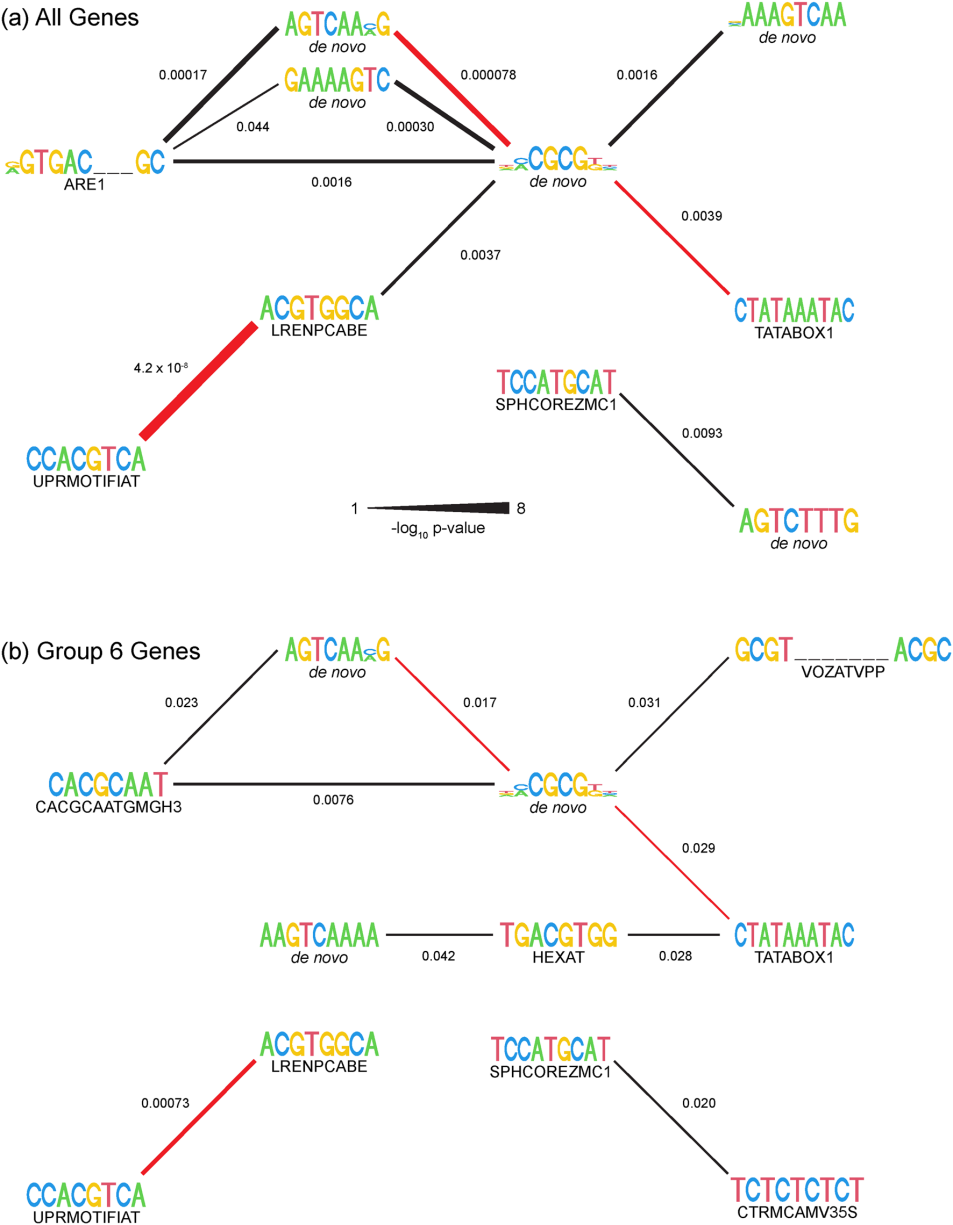
Associations were identified among upstream motifs of interest. Motifs that were closer to the TSS in induced genes and/or genes induced in resistant genotypes were tested for associations within (**a**) upstream sequences of all genes and (**b**) upstream sequences from genes in correlation Group 6. Number labels on the egdes indicate the adjusted p-value in (**a**) and the unadjusted p-value in (**b**) from Fisher’s exact tests as explained in the methods; edge thickness corresponds to the negative log_10_ of the indicated p-values. Red edges indicate connections that are present in both graphs. Motifs chosen for this display are from Tables 2 and 3, and all Fisher’s exact tests are shown in Supplementary Data 4 for (**a**) and Supplementary Data 5 for (**b**).

**Top five associated GO terms, abbreviations: C (chloroplast), CB (chlorophyll binding), CR (response to chitin), DD (damaged DNA repair), DI (DNA replication initiation), DR (defense response), ES (endomembrane system), KA (kinase activity), LC (light-harvesting complex), LT (lipid transport), M (mitochondrion), MR (mismatch repair), N (nucleus), NR (nutrient reservoir activity), P (photosynthesis), PA (polygalacturonase activity), PI (protein import into nucleus), PK (protein serine/threonine kinase activity), PM (plasma membrane), PP (protein amino acid phosphorylation), RA (response to auxin stimulus), RC (response to cold), RS (response to stress), RT (regulation of transcription), T (thylakoid), TL (chloroplast thylakoid lumen), TM (chloroplast thylakoid membrane), TF (transcription factor activity), or a dash for no associated terms;

All de novo and PLACE motifs were tested for association with each other in the upstream regions of all cacao genes (Supplementary Data 4). There were ten associations among the motifs shown in Tables 2 and 3 (Fig. 4a). These associations indicate that the motifs appeared in higher proportions together than separately. DMCGCGKH, a de novo motif associated with GO terms ‘chloroplast’ and ‘mitochondrion’, was centrally connected to 6 of the 9 other motifs with associations. The most significant association was between the motifs CCACGTCA and ACGTGGCA, PLACE motifs involved in unfolded protein response and positive light response, respectively. The motifs shown in Tables 2 and 3 were tested for association with each other among upstream sequences from Correlation Group 6 genes (Supplementary Data 5). DMCGCGKH was centrally connected again, but only to 4 of the 10 associated motifs (Fig. 4b). Three associations were present in both all genes and Group 6 genes: DMCGCGKH to AGTCAAMG, a de novo motif associated with GO terms ‘defense response’ and ‘kinase activity’; DMCGCGKH to CTATAAATAC, a PLACE motif involved in transcription initiation; and the aforementioned CCACGTCA to ACGTGGCA, which was also the strongest association identified in Group 6 genes.

The numbers of motifs of interest (as described in Tables 2 and 3) were quantified in the upstream regions of genes in Group 6 with stress or defense-related KEGG annotations (Supplementary Data 6). In general, the numbers of genes containing de novo motifs were higher in nearly all KEGG classes than those containing PLACE motifs. De novo motifs appeared more frequently than PLACE motifs in KEGG families related to disease response (e.g., LRR/NB-ARC/RK, PAMP Response, Plant-Pathogen Interactions). The de novo motifs were also prominent in ubiquitin-related genes, CAZymes, transcription factors, and genes encoding proteins with secretion signals. Five PLACE motifs showed up prominently in Group 6 genes, including TCTCTCTCT, a pyrimidine-rich motif resembling the transcription initiating Y-patch; TGACGTGG, a binding site of bZIP protein TGA1 and G-box binding factor GBF1 in Arabidopsis; ACGTGGCA, a light-responsive regulatory element; CTATAAATAC, a transcription-initiating regulatory element; and CCACGTCA, an unfolded protein response motif. PLACE motifs were frequent in ubiquitin-related genes, CAZymes, transcription factors, and genes encoding proteins with secretion signals, but to a lesser extent than de novo motifs.

**Figure 5:**
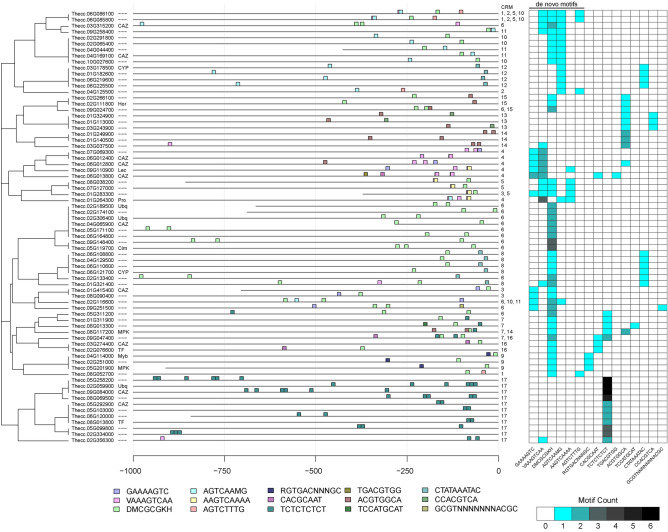
Upstream regions of genes showing putative CRMs. Upstream region diagrams (left) show positions of motifs on the sense (+) strand within 1000 bp upstream of the TSS in genes from correlation Group 6. Heatmap (right) shows number of motifs per upstream region.

To identify position-specific motif associations, or potential *cis-*regulatory modules (CRMs), the upstream regions for all group 6 genes containing motifs of interest were plotted (Supplementary Figs. S6–S18). All combinations of two motifs, or two instances of one motif, were noted. Upstream/downstream order was maintained when observing each combination, and any motifs with a majority of the sequences overlapping (e.g. VAAAGTCAA and AAGTCAAAA) were discarded as CRMs. There were 17 potential CRMs identified in group 6 (Supplementary Table S4). There were six CRMs with two motifs separated by less than 100 bp, and 10 CRMs with two motifs separated by less than 250 bp. Upstream regions displaying these CRMs were manually curated and plotted (Fig. 5, Supplementary Table S5). Among these CRMs, CAZymes contained CRMs 3, 4, 6, 11, 16, and 17, ubiquitin-related genes contained CRMs 6 and 17, MAPK signaling genes contained CRMs 7, 9 and 14, TFs contained CRMs 9 and 17, and cytochrome P450s (CYPs) contained CRMs 8 and 12 (Supplementary Table S5).

## Discussion

The seven cacao genotypes studied displayed a range in post-penetration resistance reactions to Ppal infection; four genotypes were susceptible and three were resistant. The outcome of infection in this system, i.e., susceptibility or resistance could be quantified in all seven genotypes by 72 hpi (Fig. 1c). Three of the genotypes, CCN51, Sca6 and Pound7, displayed resistance to Ppal infection, and were differentiated from the susceptible genotypes ICS1 and WFT at 48 h post-inoculation. Pound7 was previously determined to carry resistance to Ppal in pods^38,39^ which is evident in our study by lower necrosis levels, signifying reduced spread of the pathogen (Fig. 1, Supplementary Fig. S1). Similar suppression of lesion development in size was also observed in CCN51 and Sca6. Sca6 is known to have both penetration and post-penetration resistance^7,8^ and was found to be the 7^th^ most resistant clone in a leaf assay study^28^. It was surprising that CCN51 displayed post-penetration resistance in this study since the genotype is generally considered susceptible to Phytophthora infection. In our earlier study using detached leaves and zoospores to screen for penetration resistance, CCN51 displayed a susceptible phenotype^26^. In that study, Pound7 displayed a resistant phenotype and ICS1 displayed a susceptible phenotype. In a panel of 60 genotypes by Fister et al.^28^, Pound7 was the most resistant of 60 genotypes and ICS1 was used as a standard susceptible host. Iwaro et al.^8^ previously found that resistance mechanism responses to penetration were inconsistent between leaves and pods while ranking of genotypes for post-penetration resistance was consistent between leaves and pods, however they did not compare penetration resistance to post-penetration resistance. It is currently unclear why CCN51 is susceptible to Ppal penetration in leaves as previously determined^26^ and post-penetration resistant in pods as determined by the current study, but future studies may be designed to better determine these causes.

We used a gene correlation approach to identify patterns of gene expression across genotypes, identifying 34 gene correlation groups. In these groups, defense response-related gene families, including LRRs (Groups 2, 4, 12, 13, 14, 16, 17, 19, 20 and 24), PR10s (Group 6), WRKYs (Group 6), Germin-like proteins (Group 6), MAPKs (Groups 6 and 10) and ERFs (Groups 10 and 11), were found in the noted groups more than expected by random chance (Supplementary Fig. S3). These gene families are reportedly induced and associated with responses to infections in cacao and other plant species^21,26,27,40–42^. The correlation groups enriched with defense-related genes, i.e. Groups 6, 10, 11, also contain genes either from other families or with no functional annotations. Group 6 is particularly interesting as a group with 1775 genes with a strongly positive mean log_2_ fold change in all genotypes. The profile of genes in Group 6 indicates that, regardless of the susceptibility or resistance of the genotype, cacao pods activate a suite of general defense response genes when infected by Ppal.

ICS1 is an established susceptible genotype^7,23^, for example, ranking as the sixth most susceptible genotype of 60 genotypes studied in a detached leaf bioassay study^28^. In the current study, ICS1 had the lowest number of differentially expressed genes in response to Ppal infection (Fig. 2a,c; Supplementary Fig. S2b). Due to both the known susceptibility of ICS1 and the low number of differentially expressed genes, we used ICS1 as a baseline control to compare constitutive and induced expression of genes in all other genotypes. Our expression pattern profiling identified Group 6 as containing many genes with Pattern S—that is, induction of genes to a higher level as compared to ICS1—in resistant genotypes, but not in susceptible genotypes (Fig. 3). Group 6 also contained many Pattern Y genes—that is, genes induced in the genotype of interest but not to a higher level than ICS1—in both susceptible and resistant genotypes. Interestingly, while there were only 143 genes classified as Pattern S in all three resistant genotypes, there were 1521 genes classified as either Pattern S or Pattern Y in all three resistant genotypes. This suggests both differences and similarities among the molecular mechanisms controlling post-penetration resistance in CCN51, Sca6 and Pound7.

The cacao pod transcriptome shows a complex pattern of differential gene expression, which includes differential expression among genotypes of (1) constitutively expressed genes and (2) genes induced by infection which potentially participate in post-penetration resistance. Some of these genes participate in pathways associated with pathogen signal transduction, processes which lead to gene activation and enhanced gene expression^27,29,33^. An earlier study on disease resistance to black pod in cacao identified 15 consensus QTLs located on chromosomes 1, 2, 4 and 5^20^, a clear indication of the complexity of the genetic control of *Phytophthora* resistance in cacao.

Prediction of novel motifs (de novo motif discovery analysis) was conducted on the upstream promoter sequences of DEG sets in each of the seven cacao genotypes. Of the 76 motifs identified, 42 were over-represented in the upstream sequences of genes in the 34 correlation groups (Supplementary Table S3). Position analysis of motifs present in the upstream sequences of Group 6 genes revealed some motifs in similar positions within 1000 bp of the transcription start site (TSS). The boundary from − 500 to 0 bp is an adequate region to look for the majority of transcription factor binding sites laying in the proximal promoter regions as this region roughly overlaps with most potential binding sites predicted in Arabidopsis and rice^43^. In Arabidopsis, nearly two-thirds of the examined TFBSs are within the region from − 1000 to + 200 bp, and TFBSs often have a positional binding preference within that proximal region^44^. Our analysis allowed us to find de novo and known (PLACE) TFBSs that were within the range of − 1000 to − 1 bp before the TSS (Supplementary Data 1).

We identified 6 de novo and 16 PLACE motifs that were positioned close to the TSS in Ppal-induced and resistance-associated genes (Tables 2 and 3). DNA elements being positioned proximal to the core promoter allows the physical interaction between proteins binding to the elements and transcription initiation proteins binding to the core promoter^45^. Our motivations for conducting additional analyses with these motifs was as follows: (1) the motifs were enriched in correlation group 6 and were thus enriched in genes expressed to a higher level in resistant genotypes (following expression pattern S), and (2) the motifs were closer to the TSS in induced genes, in either genes induced in all genotypes (Table 2) or genes induced in resistant genotypes (Table 3). We hypothesize that motifs identified with these criteria are present represent a DNA-binding mechanism by which resistant genotypes induce defense response genes during interactions with Ppal. This work is a crucial starting point to understanding cacao-Ppal defense responses.

We quantified the presence of these motifs in defense-related KEGG families, such as calmodulin-binding proteins, plant pathogen response, MAPK signaling, TF families, ubiquitin-related, and more. The de novo motifs were more prevalent than PLACE motifs in genes with these defense-response KEGG classifications (Supplementary Data 6). There were some overlaps among these de novo motifs, e.g. GAAAAGTC, VAAAGTCAA, AGTCAAMG, and AAGTCAAAA all had the AGTC core motif that was associated with kinase activity, defense response, and response to chitin (Supplementary Fig. S4). The AGTC core is present in known motifs, including an elicitor-responsive element found in tobacco class I chitinases^46^ and an expanded W-box motif found in cotton sesquiterpene synthase genes^47^. The motifs with this shared motif are similar to a motif recognized by Whirly transcription factors (GTCAAAAA/T) that is enriched in promoters of Arabidopsis genes that are co-regulated during systemic acquired resistance^48^. The highly associated motif DMCGCGKH closely resembles a motif targeted by signal-responsive (SR) calmodulin-binding protein to activate genes involved in ethylene signaling, abscisic acid signaling, and light signal perception^49^. The Arabidopsis CAMTA3/SR1 gene is involved in drought and abscisic acid responses, and induces stomatal closure, a trait important in some plant-pathogen interactions^50^. Two of the three cacao CAMTA/SR orthologs, Thecc.02G138500 and Thecc.02G205100, were classified into correlation Groups 10 and 23, respectively. DMCGCGKH was present in the upstream sequences of Group 10 and 23 genes more than expected through random chance (Supplementary Table S3, Supplementary Fig. S4), along with other motifs with the CGCG core (ADMCGCGKHT in Groups 10 and 23, ACGCGGYK in Group 10), indicating potential co-expression of SR transcription factors with the genes they induce.

Among the PLACE motifs that were closer to the TSS in Ppal-induced and resistance-induced genes, four were prevalent in Group 6 genes associated with defense-related KEGG pathways: the TATA box (CTATAAATAC), a pyrimidine-rich motif (TCTCTCTCT), a light response motif (ACGTGGCA), and an unfolded protein response motif (CCACGTCA). The TATA box is a well-characterized regulatory binding site that is found in 29% of Arabidopsis promoters and 19% of rice promoters, clustered around − 32 bp upstream of the TSS^51,52^. The TATA box is conserved in the promoters of all Arabidopsis genes encoding PR proteins and most rice PR protein-encoding genes^53^. In cacao, 7473 out of 27,379 genes (27.3%) have a TATA box or TATA-like motif in the region within 100 bp upstream of the TSS, with 3668 (13.4%) of genes having a TATA box or TATA-like motif clustered around − 32 bp (Supplementary Data 7, Supplementary Fig. S19). The TCTCTCTCT motif is identified in PLACE as a CaMV 35S promoter, but resembles the Y-patch, a pyrimidine-rich regulatory element required for general gene induction present in 50% of rice genes^52^. The TGACGTGG motif is a binding site of Arabidopsis TGA subfamily of bZIP transcription factors like TGA1 that was previously found to be induced preferentially by infection with Cauliflower mosaic virus and *Botrytis cinerea* in Arabidopsis^54^. While the PLACE motifs of interest were less prevalent than de novo motifs in the upstream region of genes with defense-related KEGG annotations, the identification of these motifs is still important and, along with the de novo-identification of motifs, provides a solid foundation in understanding gene transcriptional regulatory mechanisms in the cacao-Ppal interaction.

We identified potential *cis-*regulatory modules (CRMs), or clusters of DNA elements that combine to have regulatory effects, by examining the positions of our motifs of interest in Group 6 genes (Supplementary Figs. S6–S18). Seventeen candidate CRMs were identified in the upstream regions of Group 6 genes (Supplementary Tables S4–S5; Fig. 5). CRMs 6 and 17 were the most common in Group 6 and consisted of repeats of the same motifs appearing mostly between − 1 and − 250 nt before the TSS—DMCGCGKH in CRM6 and TCTCTCTCT in CRM17. Both CRMs were present in the upstream regions of CAZymes and ubiquitin-related genes, suggesting a role in cellular metabolism. Among the CAZymes with these modules were a predicted galacturonosyltransferase (Thecc.04G065900) and a predicted O-glycosyl hydrolase family 17 (Thecc.05G292900), enzymes involved in pectin biosynthesis^55^ and cell wall organization^56^. Because the plant cell wall is the direct interface between plant and pathogen^57^, pectin biosynthesis and cell wall organization are likely to be important processes in the cacao response to *Phytophthora*.

CRM4 was another module with close repeats of a single motif, VAAAGTCAA, that appeared in the upstream region of three Group 6 CAZyme genes putatively encoding berberine-interacting proteins, a FAD-binding and berberine bridge enzyme-like (BBE) domain-containing protein (Thecc.06G012400) and two Tetrahydroberberine oxidases (Thecc.06G012800 and Thecc.06G013800). While BBE-like enzymes are generally in alkaloid biosynthesis, genes encoding BBE-like proteins have been earlier identified as monolignol reductases in Arabidopsis, playing a role in monolignol metabolism and lignin formation^58^. Arabidopsis BBE-like proteins have a complex role in pathogen defense. Several BBE-like proteins degrade damage associated molecular patterns (DAMPs)^59,60^. In some cases, this leads to host susceptibility by impairing the elicitor activity of DAMPs, but in other cases enhances defense response due to removal of an easy-to-acquire carbon source. The ancestral role of BBE-like proteins, as identified in the bryophyte *Physcomitrella patens*, is cellobiose oxidase, a step of cellulose degradation^61^. If the genes identified here have roles in cacao cell wall turnover, they are likely interesting targets for further research into cacao-*Phytophthora* interactions.

Another CAZyme likely involved in defense against oomycete pathogens is the Class EP3 chitinase Thecc.04G169100, which contained CRM11 (DMCGCGKH followed by ATGCAAMG, within 50 nt in this case). Chitinases are known to be induced during pathogen infection, including *Phythopthora* infection in cacao^21,62^ and transient expression of chitinase in cacao leaves increased resistance to *Phytophthora tropicalis*^27^.

This study separated the seven genotypes studied into two groups based on their response to *P. palmivora*: susceptibility (ICS1, WFT, Gu133, Spa9) and resistance (CCN51, Sca6 and Pound7). By using RNA-Seq to profile the transcriptomes, we found that the shared response was greater among resistant genotypes than susceptible. We further identified groups of co-expressed genes, including 1755 genes in Group 6, which were induced in all genotypes, but to a higher level in resistant genotypes. This allowed us to identify gene and promoter element candidates for resistance breeding and for further analyses in efforts to understand early components of post-penetration defense-related gene activation in cacao. Both novel and known sequence motifs close to the TSS in upstream regions of cacao Ppal-induced genes were characterized. The identification and characterization of motifs and motif interactions (i.e., CRMs) is crucial to understanding gene-regulation in cacao pod tissues and how gene networks are regulated in response to pathogens like Ppal. Characterization of motif positioning within promoters of defense response QTLs and genes can be an effective strategy for identifying critical regulatory components of a plant’s defense-response transcriptome. This resource is therefore provided to support future and ongoing efforts to breed Ppal-resistant cacao.

## Materials and methods

### Pod sampling

Three-month-old pods were harvested from cacao trees clonally propagated in a greenhouse of genotypes Pound7, CCN51, Sca6, ICS1, WFT, Spa9 and Gu133 in replicates of three pods from separate, individual trees of each genotype. Sampling was random based on the availability of pods on individual trees of each genotype with replications initiated on separate days. These trees are maintained in the USDA-ARS, Beltsville, MD cacao greenhouse at ambient relative humidity (approximately 60%) and a minimum day length of 12 h using a supplemental light intensity of at least 325 μmol/m^2^/s. All experimental research conducted on plants complied with relevant institutional (USDA-ARS), national, and international guidelines and legislation.

### *Phytophthora palmivora* inoculum preparation

The Ppal isolate used, Gh-ER1349, was previously isolated from BPR-infected cacao in Ghana^63^ and maintained on a clarified V8 juice (CV8) agar plate at 18 °C. Ppal was grown on a CV8 agar plate (90 mm) for 7 days under constant dark at 25 °C and then transferred to constant light (200 lx) for 4–5 days.

### Pod inoculation

Pods were cut into half and rectangular pieces and wounded with a no. 1 cork borer (5 mm diameter). Inoculations were performed with Ppal plugs taken from CV8 plates using a plugger of 153 mm length × 6 mm diameter (Spectrum™ transfer tube 190195) with plugs placed to the left end of the pod piece so that spread could be monitored. Uninoculated CV8 agar plugs were used for control pod pieces. We inoculated 6 pod pieces of each genotype on each replicate, with 5 total replicates conducted. Necrosis levels were measured at 24, 48 and 72 hpi by measuring spread from the point of inoculation. Pod piece sizes were chosen to facilitate RNA extraction for later analysis and the final time point for necrosis measurements was chosen to allow lesion measurement before the lesion covered the entire small pod piece. The average necrotic lesion sizes per pod piece were plotted using the boxplot and stripchart functions in R base version 1.4.3 (R Core Team: https://www.r-project.org/). Pairwise F-tests via the R function var.test were used to confirm homogeneity of variance among all samples. To determine if differences among genotypes per timepoint existed, an ANOVA was conducted via the aov function in R. A post-hoc Student–Newman–Keuls test, via the SNK.test function in the R library agricolae version 1.3–5, was conducted to determine statistical grouping^64^.

### RNA extraction

For each cacao variety inoculated, three of the five 48 h pathogen infection assay replicates were chosen at random for total RNA extraction for subsequent RNA-Seq analysis. A total of twenty-one infected (three replicates for seven genotypes) and twenty-one control (three replicates for seven genotypes) pod tissue pieces were finely grounded in mortar and pestle, and total RNA extraction was performed as previously described^65^. RNA purity and concentration were assessed using an Axygen Gel Documentation system and a NanoDrop™2000 spectrophotometer (Thermo Fischer Scientific, Waltham, MA, United States) and were in the required quality range (1.8–2.0) of A_260/280_ absorbance ratio.

### mRNA isolation, cDNA synthesis and sequencing

Isolation of mRNA, cDNA synthesis, and library assembly and sequencing were outsourced and carried out by BGI Genomics (Hong Kong). Initial RNA quality was verified using the RNA integrity number obtained using an Agilent Technologies 2100 Bioanalyzer. The 42 libraries were prepared for DNBSEQ platform following proprietary BGI Genomics library preparation methods. The libraries were amplified with Phi 29 DNA polymerase to create DNA nanoballs, which were loaded into a patterned nanoarray. Paired-end reads of length 150 bp per mate pair were generated using combinatorial Probe-anchor Synthesis.

### RNA-Seq analysis

RNA reads from RNA-Seq libraries ranging from 40.7 M to 47.9 M raw sequence reads in FASTQ format were trimmed to remove adapter sequences using BBDUK version 37.58, using adapters.fa with parameters ktrrim = r, k = 23, mink = 11, hist = 1, tpe, tbo^66^. Trimmed reads were purged of Ppal sequences by aligning them to the Ppal isolate Gh-1349 reference genome^67^. Reads that survived purging were aligned to the cacao Matina 1–6 reference genome v2.0 using HISAT2 version 2.1.0^37,68^. Tabulated raw counts from each CDS were obtained from the HISAT2 alignment. Raw counts were normalized, and differential gene expression analysis was conducted using DESeq2^69^ available in the Galaxy pipeline (Galaxy Version 2.11.40.1). Genes with fdr-adjusted p-values less than 0.05 were considered differentially expressed.

### Gene correlation matrix construction and analysis

A gene correlation matrix was constructed, with all calculations conducted in R base version 4.1.3 (R Core Team: https://www.r-project.org/). The gene correlation matrix was constructed based on a modified version of the ‘regulatory association network’ described by Ambavaran et al*.*^70^. First, the raw RNA-Seq reads were normalized to reads per million per sample replicate. Normalized reads were log_2_ transformed to produce the gene expression matrix; prior to log transformation, 1 was added to all values to prevent infinity as output on values with zero reads. Genes were retained after normalization only if they had 10 or more reads in at least 5 of 42 of sample replicates prior to normalization. A correlation matrix comparing every gene–gene pair was produced with the cor function in R with method = "pearson". A distance matrix was produced with the as.dist function in R on 1—correlation. Hierarchical clustering was performed on the distance matrix with the hclust function in R with method = "complete".

The optimal tree-cutting height was determined by inspecting both the number of groups produced and the mean correlation per group at different cutting heights. The tree was cut at height = 1.5 using the cutree function in R. Odds ratios of gene families per correlation group were estimated using 2 × 2 contingency tables and the fisher.test function in R with alternative = "two.sided" to test the alternative hypothesis: the odds ratio is not equal to 1. For each gene family in each correlation group, the corresponding contingency table contained the numbers of genes (1) in group, in gene family, (2) in group, not in gene family, (3) out of group, in gene family, and (4) out of group, not in gene family. P-values were adjusted for false discovery rate per gene family with the p.adjust function in R with method = "fdr". A significance threshold of ≤ 0.05 was used for adjusted p-values. Heatmaps of mean log_2_ fold change per group and odds ratios were plotted with the heatmap.2 function in the R library gplots version 3.1.3 (CRAN: https://cran.r-project.org/web/packages/gplots/index.html).

### Expression pattern profiling

For each genotype except ICS1, three comparisons were conducted via DESeq2 version 1.34.0^69^: (1) ICS1 mock vs. genotype mock, (2) ICS1 treatment vs. genotype treatment, and (3) genotype treatment vs. genotype mock. Genes were considered up- or down-regulated if the log_2_ fold change was positive or negative, respectively, and the FDR-adjusted p-value was ≤ 0.05. All 27 possible patterns of expression for these three comparisons were determined and the numbers of genes matching each pattern for each genotype were quantified. A pattern was classified as "dominant" within a correlation group if the number of genes in the correlation group that matched the pattern was either (a) ≥ 100 genes or (b) ≥ 10% of the total number of genes in the correlation group.

### Upstream DNA motif analysis

Sequences 1000 bp upstream of predicted transcription start sites (TSS) from the cacao Matina 1–6 reference genome v2.0 annotation were used for upstream DNA motif analysis^37^. Upstream regions that were interrupted by a coding sequence from another gene were truncated to prevent the discovery of protein-coding motifs. Motifs were discovered with STREME in the web version of the MEME Suite, version 5.4.1^71,72^. The sequences used for discovery were the sequences 1000 bp upstream of the predicted transcription start site of genes differentially expressed from each comparison (i.e. ICS1 up-regulated, ICS1 down-regulated, WFT up-regulated, etc.), with the control sequences being the upstream sequences from genes not differentially expressed in that comparison. All discovered motifs with unadjusted p-value ≤ 0.05 were retained for additional analysis. Association with gene ontology terms was determined with GOMo in the web version of the MEME Suite with the "Arabidopsis" database, which includes upstream sequences and gene ontology annotations from the five plant species *Arabidopsis thaliana*, *Oryza sativa*, *Populus* trichocarpa, *Sorghum* brachupodium, and Brachypodium distachyon^72,73^. To determine if a motif appeared in each correlation group more than expected through random chance, 2 × 2 contingency tables were prepared with counts of the numbers of upstream sequences (1) in group with motif, (2) in group without motif, (3) not in group with motif, and (4) not in group without motif. These contingency tables were used with the fisher.test function in R with alternative = "two.sided" to test the alternative hypothesis: the odds ratio is > 1, and p-values were adjusted with the p.adjust function with method = "fdr". A significance threshold of ≤ 0.05 was used for adjusted p-values. The layout of the graph was determined with the R package iGraph^74^. Sequence logos were drawn in R with the Bioconductor package seqLogo (Bioconductor: https://bioconductor.org/packages/release/bioc/html/seqLogo.html).

To determine whether motifs were positioned closer to the transcription start site in induced genes, genes were classified as induced (up-regulated in at least 4 of 7 varieties) or non-induced (not up-regulated in at least 4 of 7 varieties) and mean positions relative to the transcription start site were calculated for each motif. This analysis included both de novo discovered motifs and known motifs from the PLACE DB^75^. Variances of mean positions were compared for motifs in both induced and not induced genes using the var.test function in R. Differences between mean positions of motifs in induced and not induced genes were detected using the t.test function in R, with alternative = "greater" and var.equal dependent on the var.test p-values (i.e. with p ≤ 0.05, var.equal = FALSE for Welch’s t-test, and for p > 0.05, var.equal = TRUE for Student’s t-test). Position comparison was repeated for genes induced in resistant genotypes (up-regulated in at least 1 resistant genotype) vs. not induced in resistant genotypes (not up-regulated in any of the resistant genotype). The results of all motif position tests are in Supplementary Data 2 for induced genes and Supplementary Data 3 for genes induced in resistant genotypes.

Associations among motifs were discovered by comparing every motif to every motif. For every comparison, a contingency table was produced with the following: (1) number of upstream sequences with both motifs, (2) number of upstream sequences with motif 1 only, (3) number of upstream sequences with motif 2 only, and (4) number of upstream sequences with neither motif. These contingency tables were used with the fisher.test function in R with alternative = “greater” to test the alternative hypothesis: the odds ratio is > 1, and the resulting p-values were adjusted with the p.adjust function with method = “fdr”. Associations among motifs of interest in group 6 were discovered using a similar manner but with no p-value adjustment due to the lower number of tests. The results of all association tests are in Supplementary Data 4 for all comparisons in all genes and Supplementary Data 5 for comparisons of interest in group 6 genes. Graphs were plotted based on significant associations (adjust p-value ≤ 0.05 or p-value ≤ 0.05 for all associations and group 6 associations, respectively) after removing associations between two motifs with a majority of sequence overlap.

Position diagrams were generated for the upstream regions of all group 6 genes and motifs of interest. Genes with 0 or 1 motif were removed. The remaining genes were clustered based on motif frequency using the heatmap.2 function in the R library gplots version 3.1.3 (Warnes et al. 2022) and motif occurrences were plotted (Supplementary Figs. S6–S18). The upstream region diagrams were manually inspected to discover potential *cis-*regulatory modules (Supplementary Table S4). Genes displaying potential modules were chosen (Supplementary Table S5) and the upstream regions were clustered and plotted as described.

### KEGG analysis

KEGG^76,77^ annotations used in this publication were previously classified as described in Baruah et al.^26^.

### Supplementary Information

Supplementary Information 1.

Supplementary Information 2.

Supplementary Information 3.

Supplementary Information 4.

Supplementary Information 5.

Supplementary Information 6.

Supplementary Information 7.

Supplementary Information 8.

## Data Availability

The datasets generated and/or analysed during the current study are available in the NCBI Sequence Read Archive repository, BioProject accession PRJNA971242.
