# Supplementary material for: Cacao pod transcriptome profiling of seven genotypes identifies features associated with post-penetration resistance to Phytophthora palmivora
Source: Sci Rep. 2024 Feb 20;14:4175. doi: 10.1038/s41598-024-54355-8 (PMC10879190; doi:10.1038/s41598-024-54355-8)
Supplement: Supplementary file 8 — Supplementary Information 8. [file 41598_2024_54355_MOESM8_ESM.docx]

**Supplementary Information**

**Transcriptome profiling identifies gene expression patterns and promoter motifs associated with post-penetration resistance to *Phytophthora palmivora* in pods of *Theobroma cacao* genotypes**

Indrani K. Baruah^1*^, Jonathan Shao^2^, Shahin S. Ali^1,3^, Martha E. Schmidt^1^, Lyndel W. Meinhardt^1^, Bryan A. Bailey^1^, Stephen P. Cohen^1*+^

^1^U.S. Department of Agriculture-Agricultural Research Service, Sustainable Perennial Crops Laboratory, Beltsville, MD 20705

^2^U.S. Department of Agriculture-Agricultural Research Service, Office of the Area Director, Beltsville, MD 20705

^3^ATCC (American Type Culture Collection), Gaithersburg, MD 20877

^*^These authors contributed equally

^+^Author to whom correspondence should be addressed: [Stephen.Cohen@usda.gov](mailto:Stephen.Cohen@usda.gov)


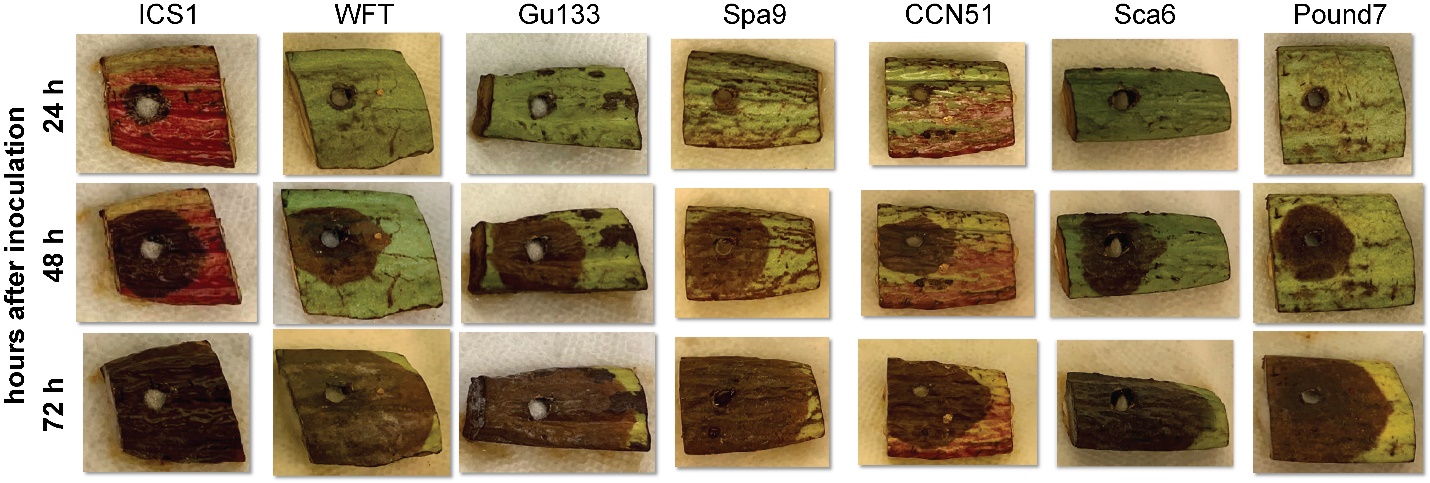


**Supplementary Figure S1. Representative necrosis symptoms for seven Ppal-inoculated cacao genotypes.**


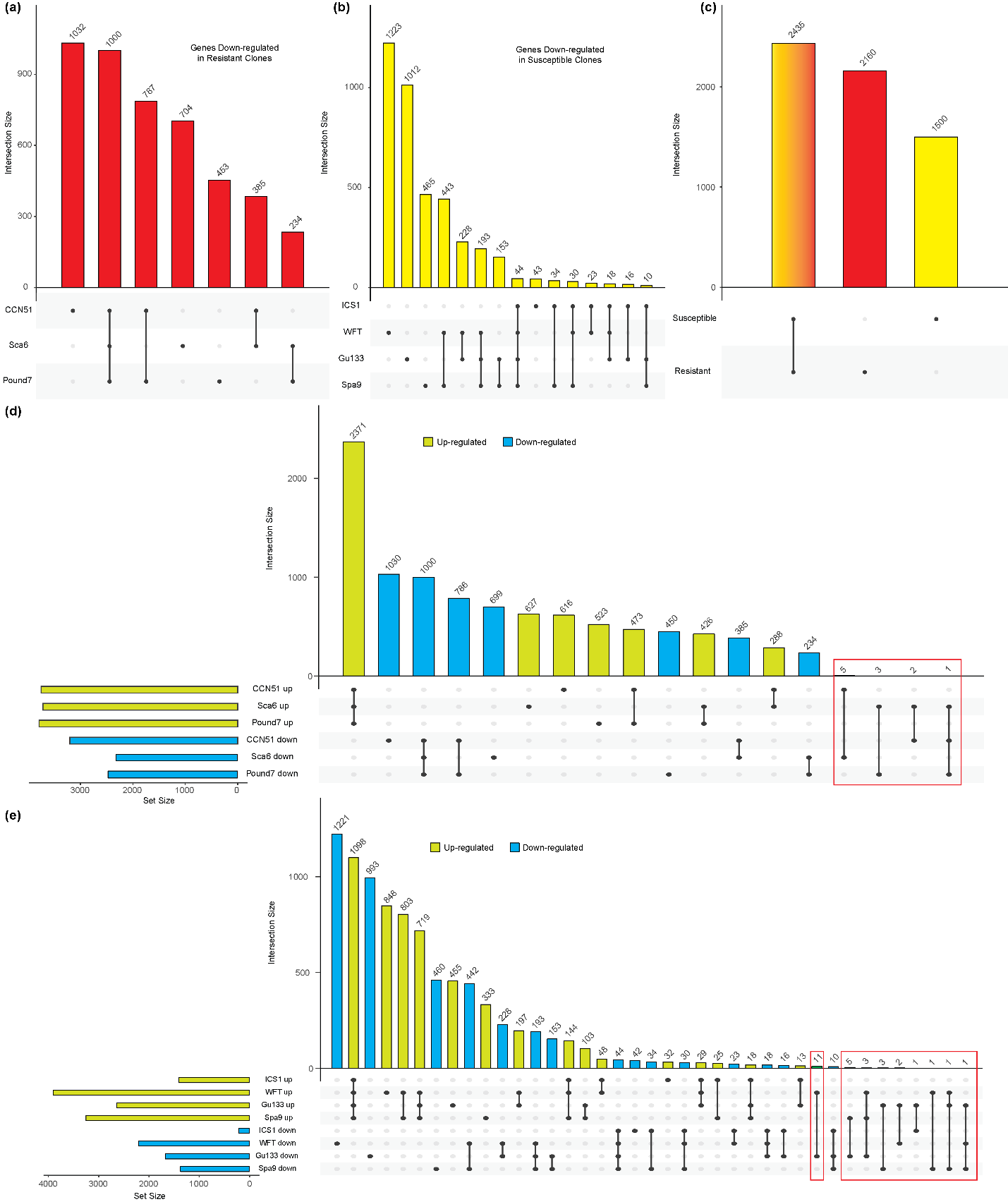


**Supplementary Figure S2. Addition differential gene expression analysis results.** Overlap in numbers of down-regulated genes in (a) resistant and (b) susceptible genotypes. (c) Overlap in numbers of genes down-regulated in at least one susceptible and/or at least one resistant genotypes. The overlap of genes regulated is shown in (d) all resistant genotypes and (e) all susceptible genotypes.


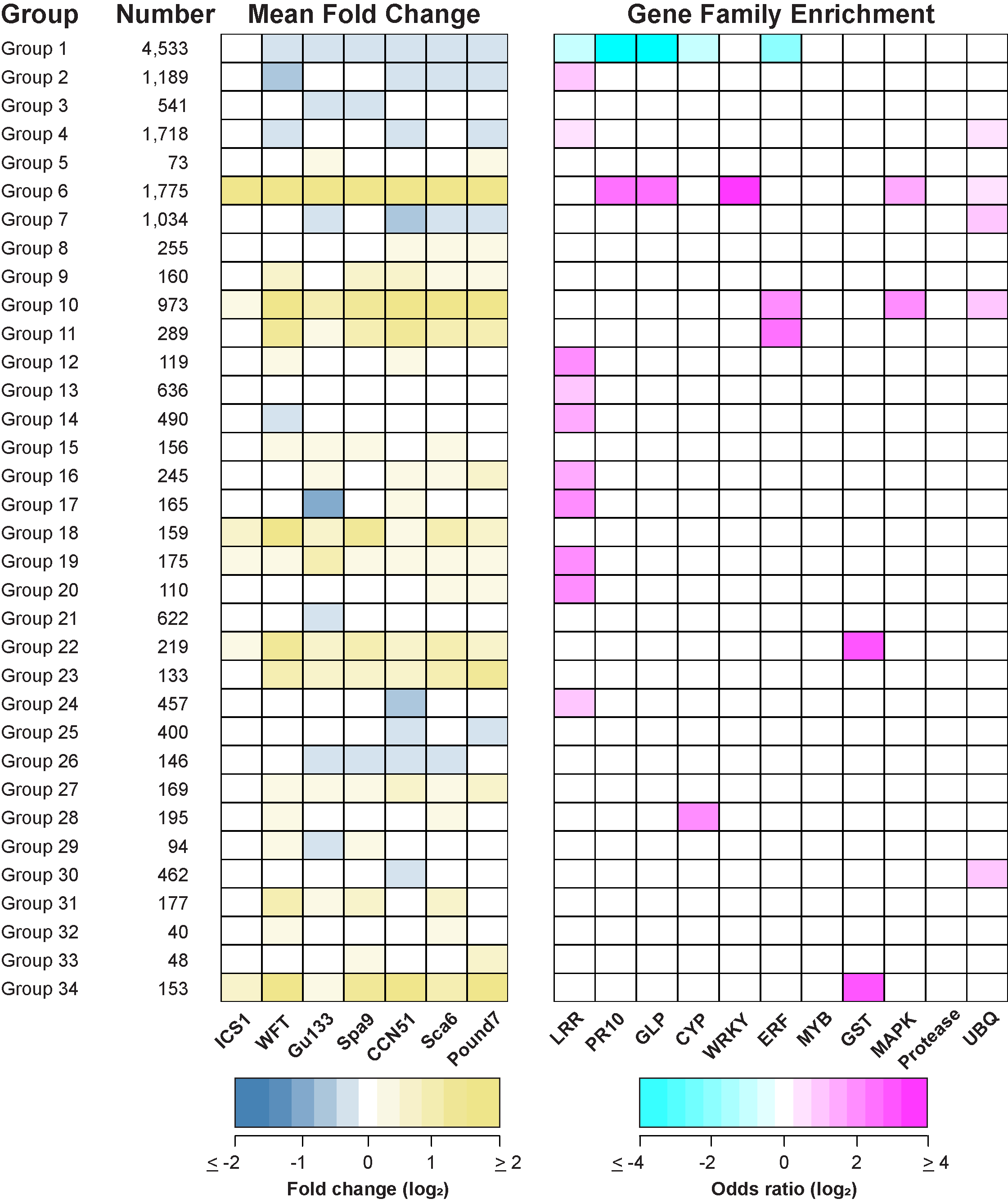


**Supplementary Figure S3. Counts of genes, mean fold changes, and enrichment of defense-related KEGG annotations within correlation groups.**


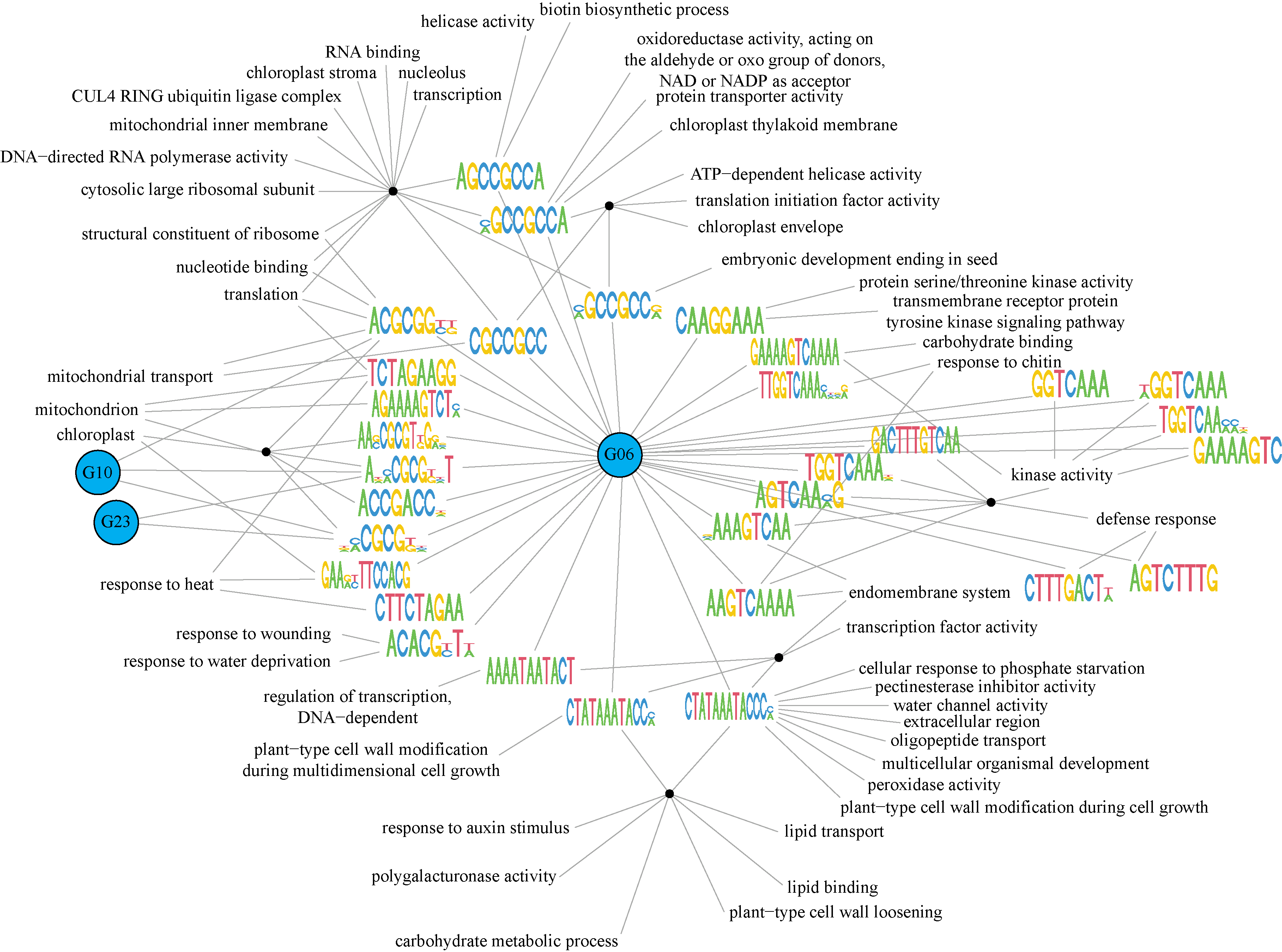


**Supplementary Figure S4. Graph of de novo motifs.** Nodes of the graph are de novo discovered motifs (sequence logos), correlation groups (blue circles), and associated GO terms. Edges among motifs and correlation groups show enrichment of motifs within the groups. Edges among motifs and GO terms show association of terms with the motifs. Unlabeled nodes (black circles) simplify the graph by showing associations common among multiple motifs.


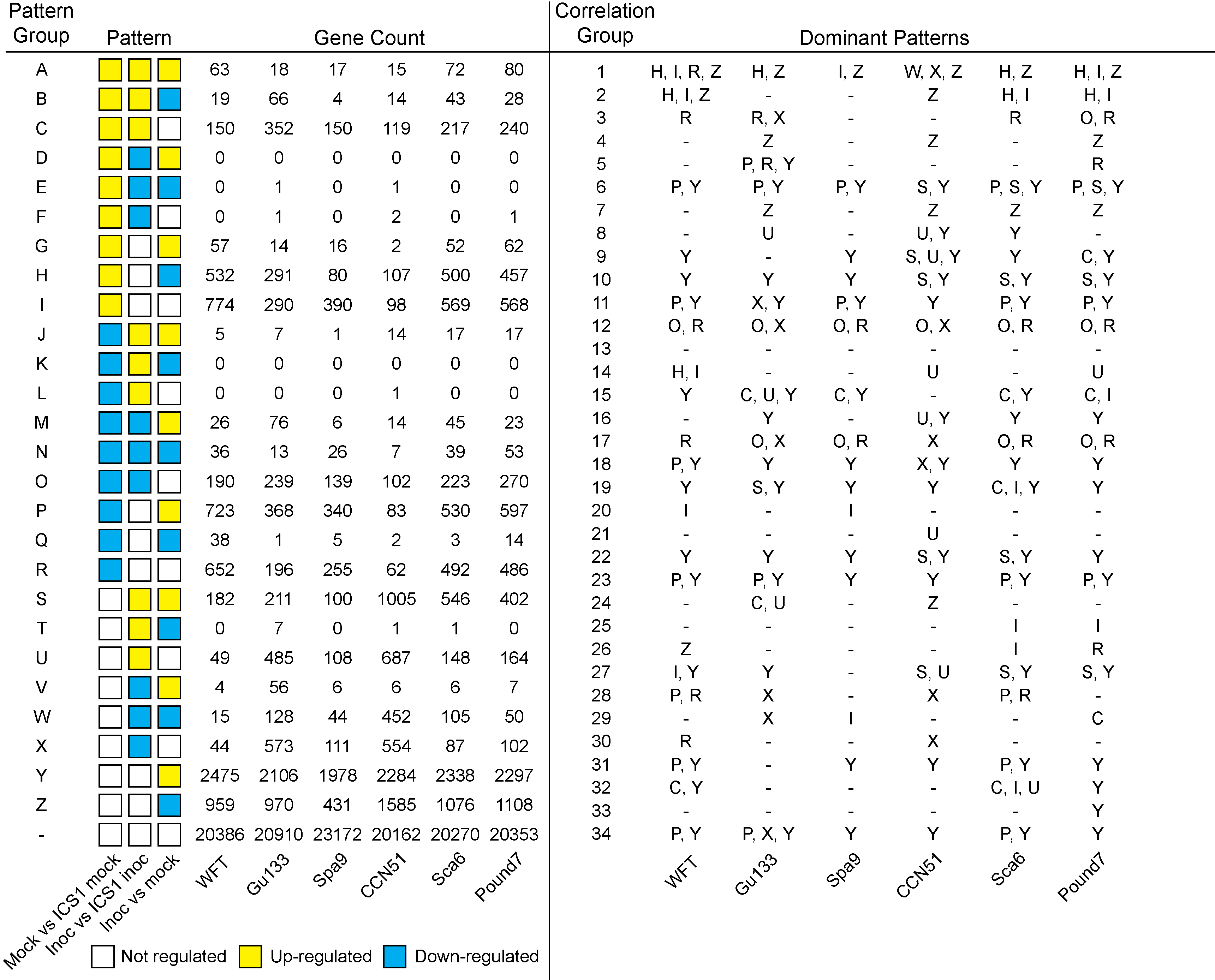


**Supplementary Figure S5. Gene expression pattern profiling of all genes.** (left) All gene expression patterns for all genotypes. (right) All correlation groups with patterns from the left panel making up >100 genes or >10% of total genes within the correlation group.


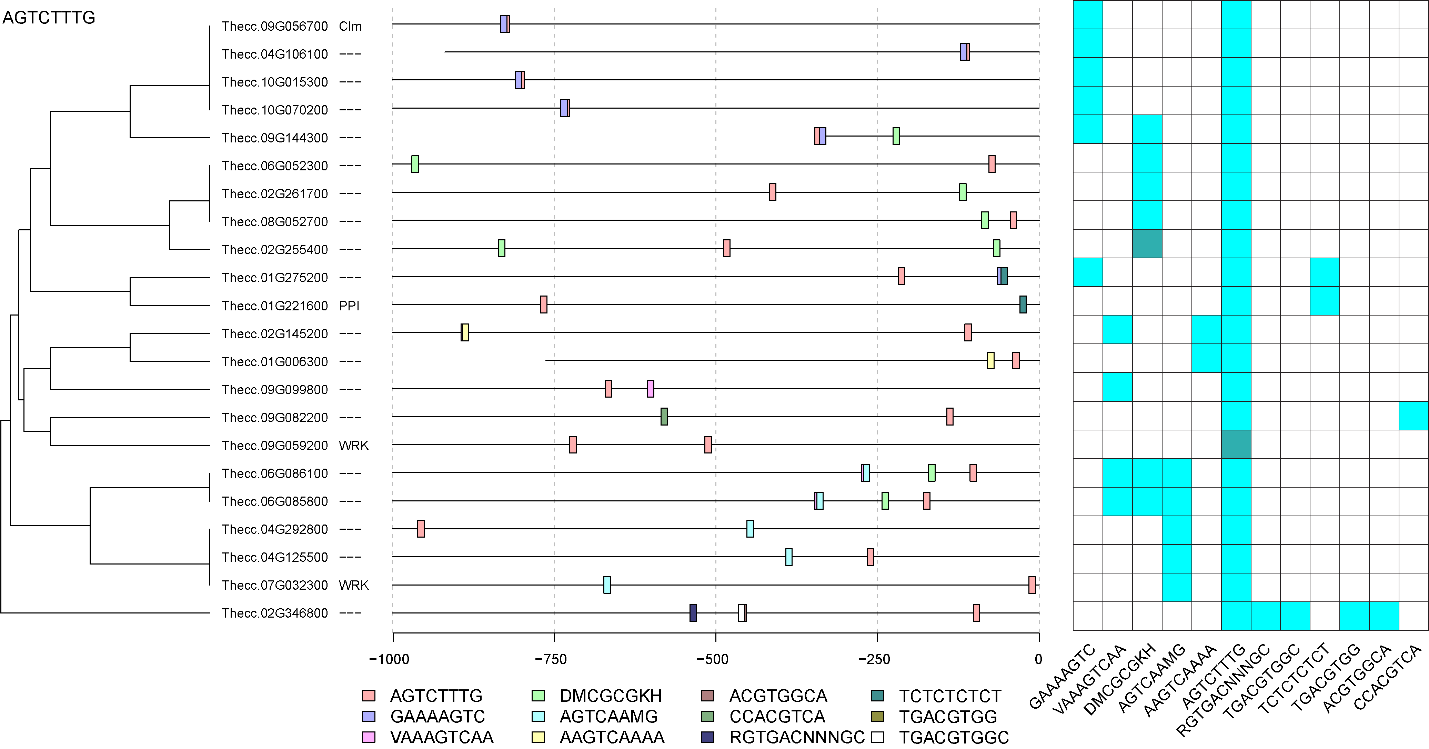


**Supplementary Figure S6. Upstream region diagrams of Group 6 genes with the *de novo* motif, AGTCTTTG.** Upstream sequences are right-aligned to correspond to the predicted TSS. Heat maps indicate the frequency of all motifs of interest per upstream sequence (white = 0, cyan = 1, teal = 2).


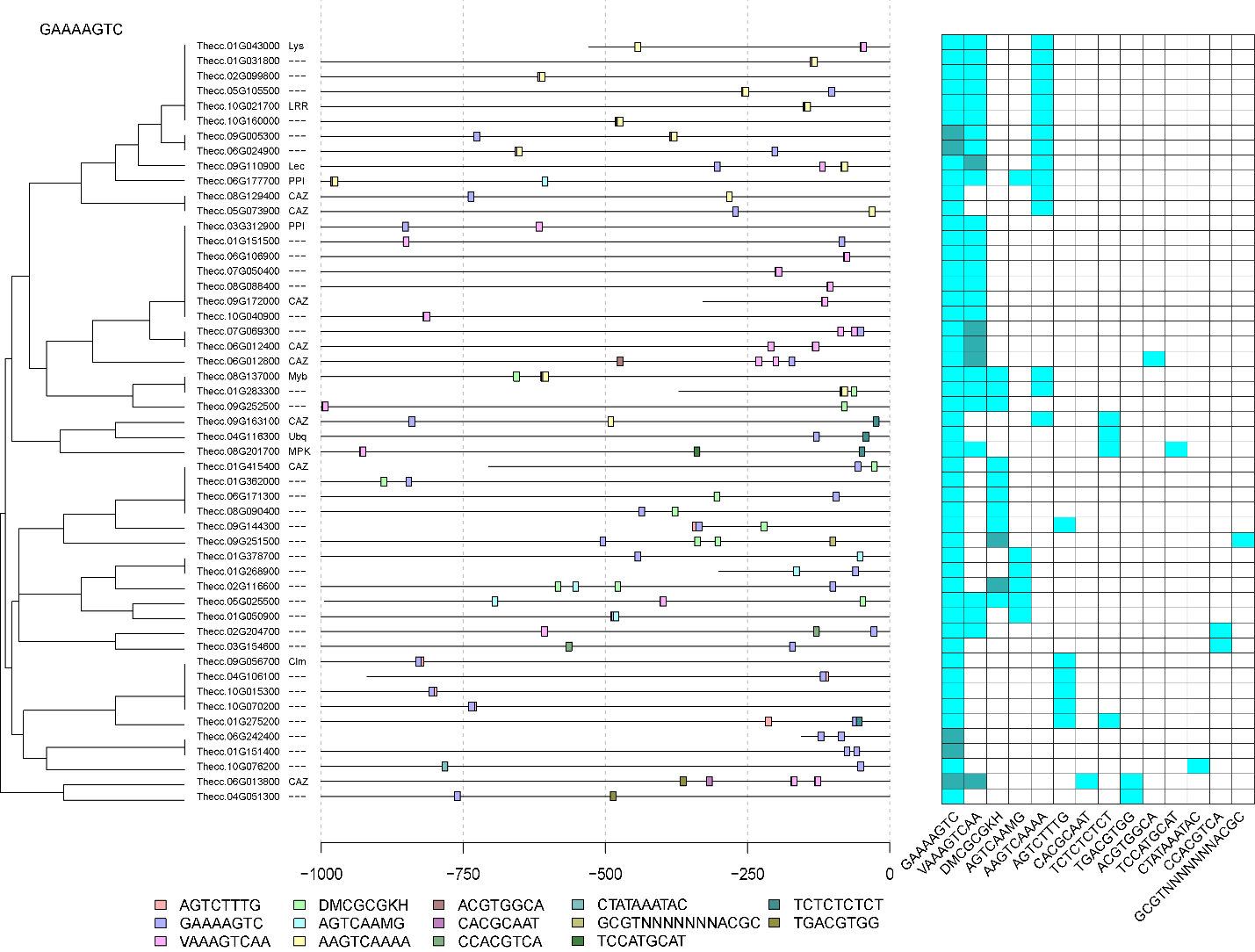


**Supplementary Figure S7. Upstream region diagrams of Group 6 genes with the *de novo* motif, GAAAAGTC.** Upstream sequences are right-aligned to correspond to the predicted TSS. Heat maps indicate the frequency of all motifs of interest per upstream sequence (white = 0, cyan = 1, teal = 2).


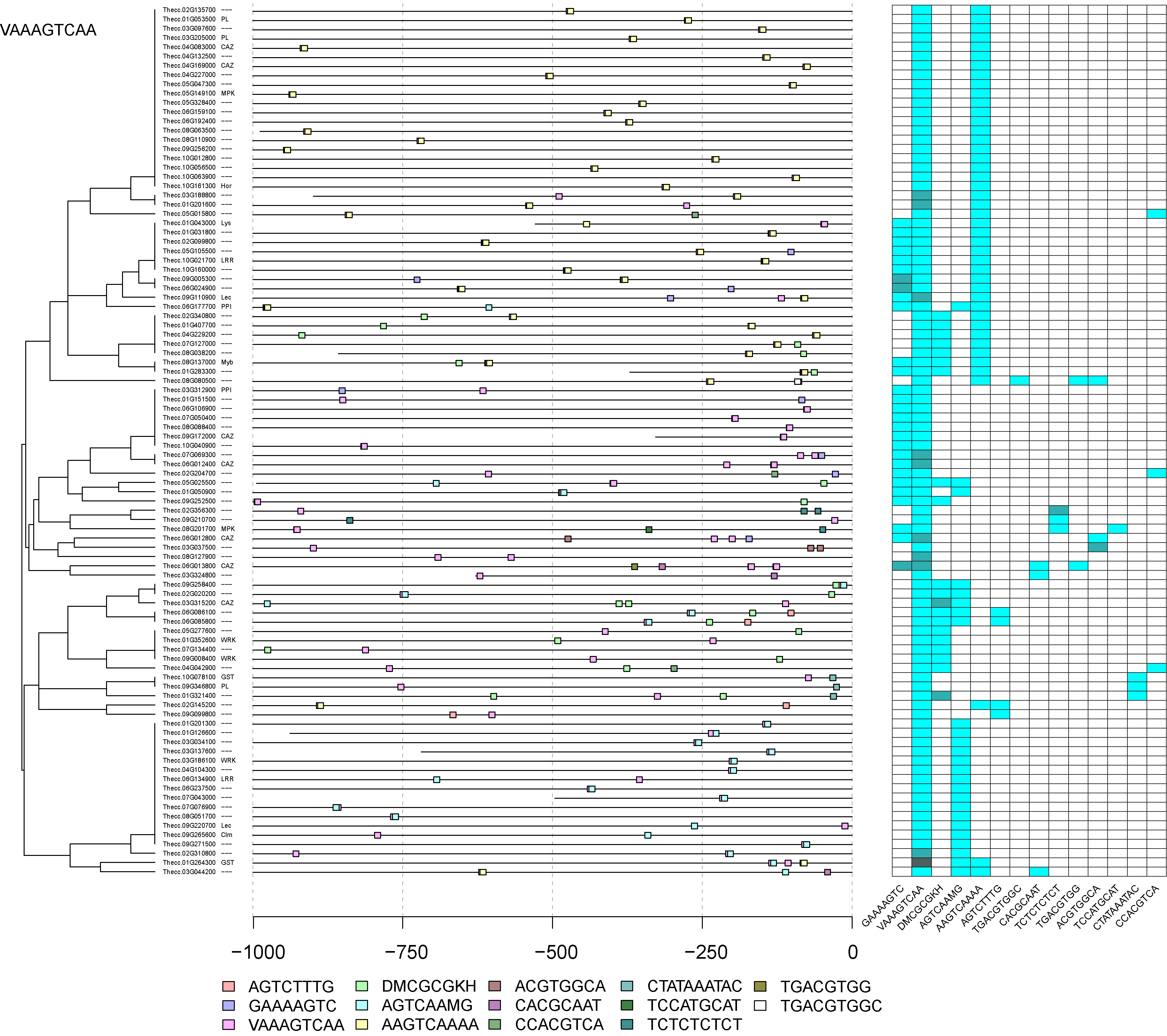


**Supplementary Figure S8. Upstream region diagrams of Group 6 genes with the *de novo* motif, VAAAGTCAA.** Upstream sequences are right-aligned to correspond to the predicted TSS. Heat maps indicate the frequency of all motifs of interest per upstream sequence (white = 0, cyan = 1, teal = 2, dark teal = 3).


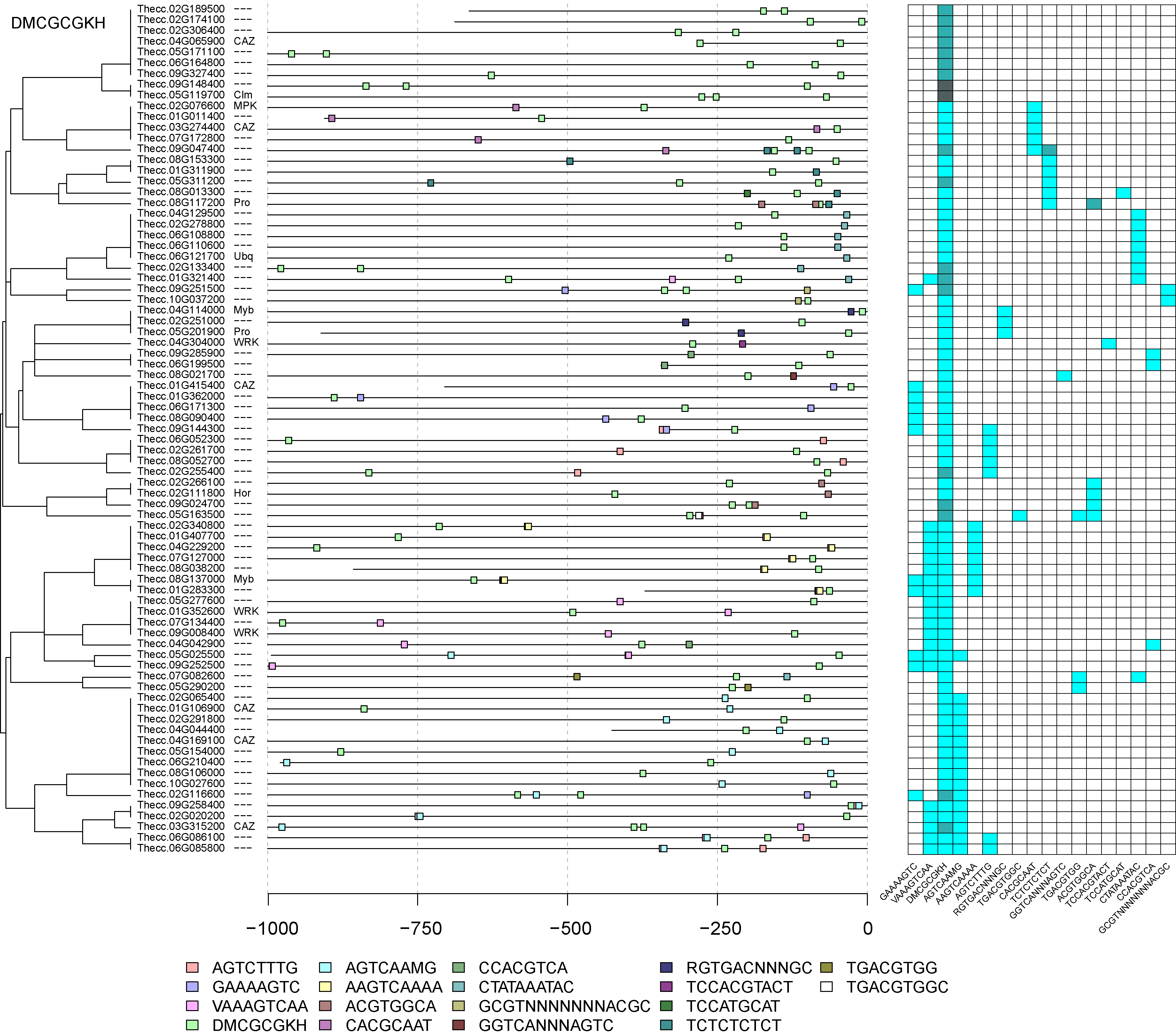


**Supplementary Figure S9. Upstream region diagrams of Group 6 genes with the *de novo* motif, DMCGCGKH.** Upstream sequences are right-aligned to correspond to the predicted TSS. Heat maps indicate the frequency of all motifs of interest per upstream sequence (white = 0, cyan = 1, teal = 2, dark teal = 3).


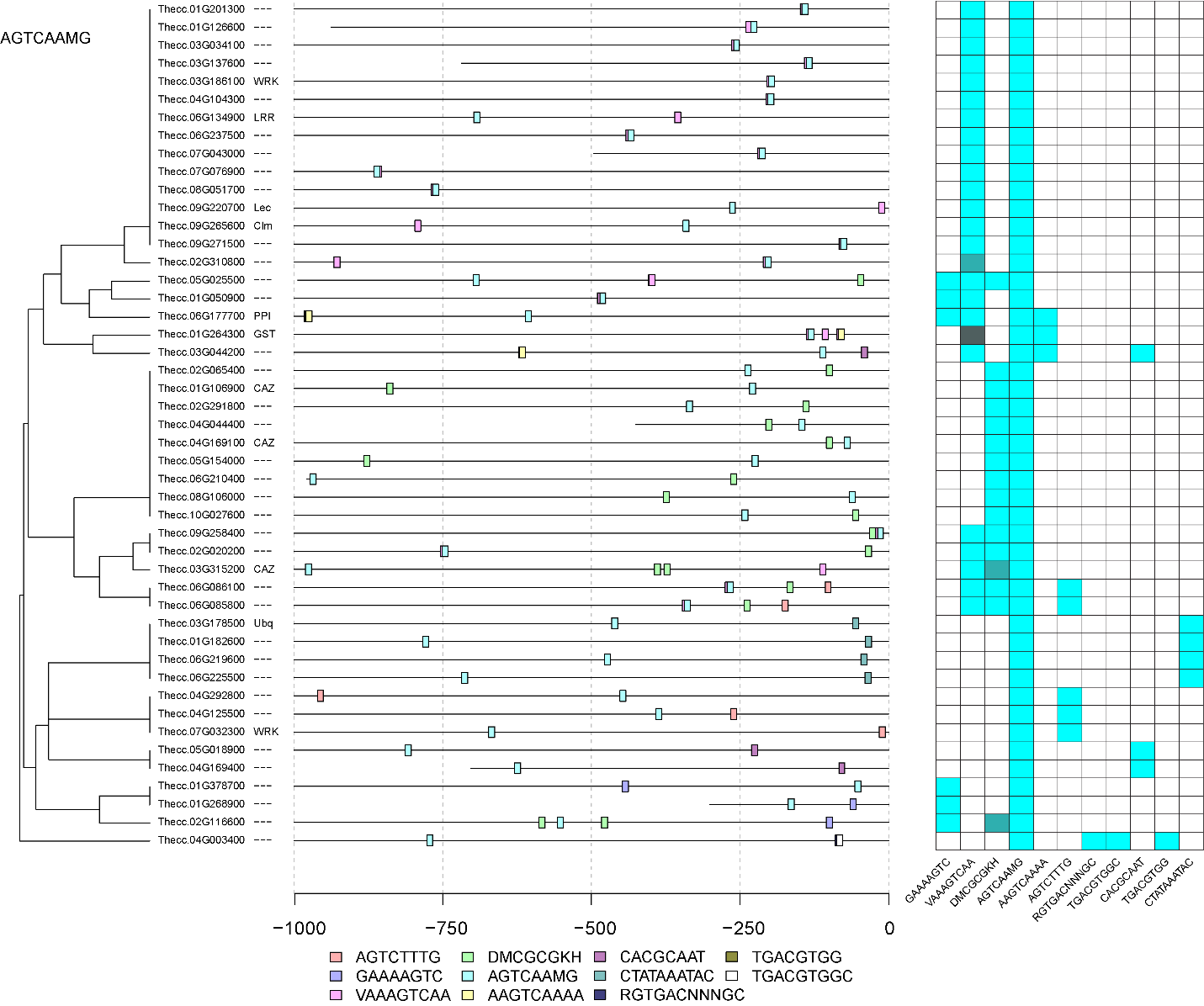


**Supplementary Figure S10. Upstream region diagrams of Group 6 genes with the *de novo* motif, AGTCAAMG.** Upstream sequences are right-aligned to correspond to the predicted TSS. Heat maps indicate the frequency of all motifs of interest per upstream sequence (white = 0, cyan = 1, teal = 2, dark teal = 3).


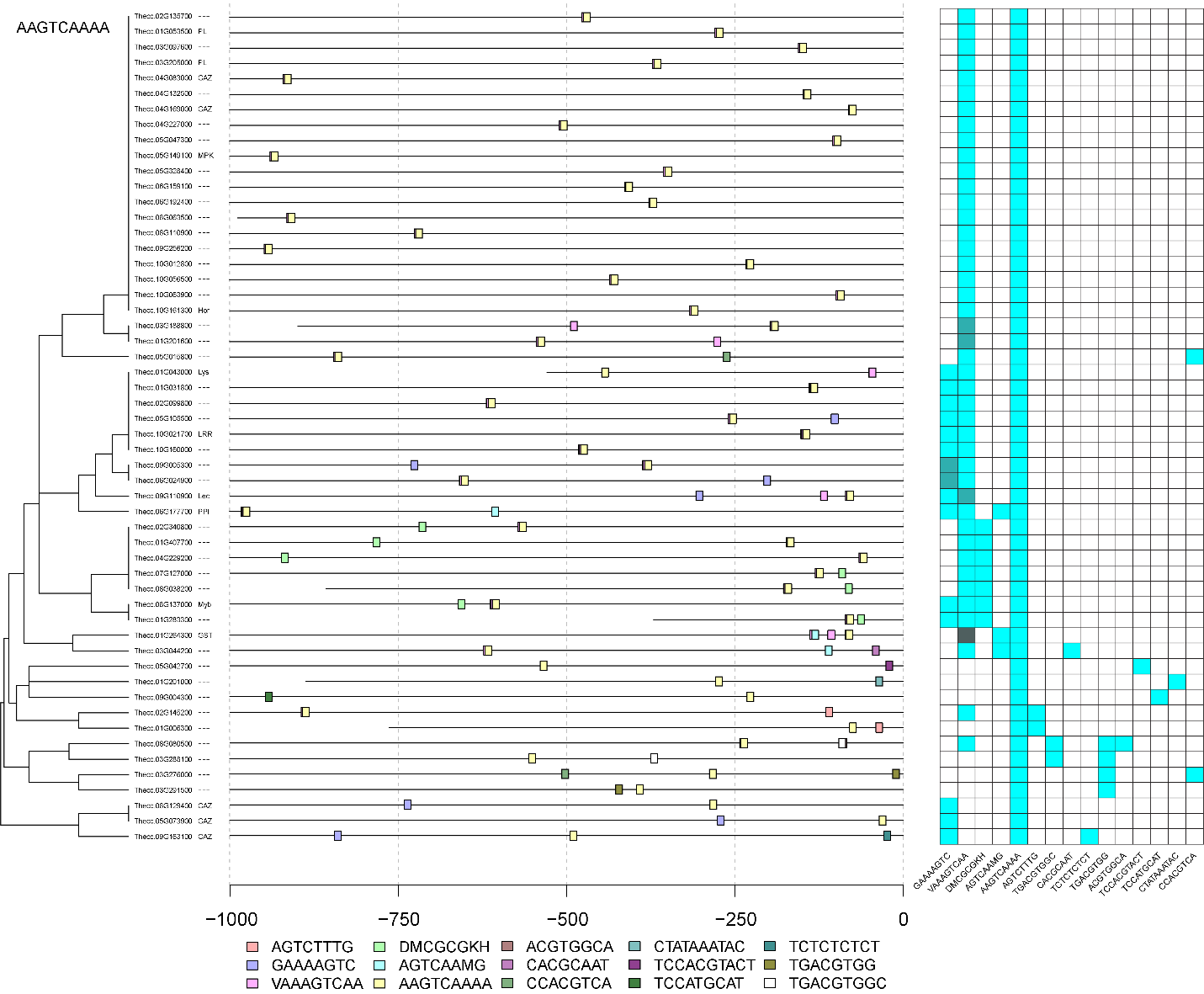


**Supplementary Figure S11. Upstream region diagrams of Group 6 genes with the *de novo* motif, AAGTCAAAA.** Upstream sequences are right-aligned to correspond to the predicted TSS. Heat maps indicate the frequency of all motifs of interest per upstream sequence (white = 0, cyan = 1, teal = 2, dark teal = 3).


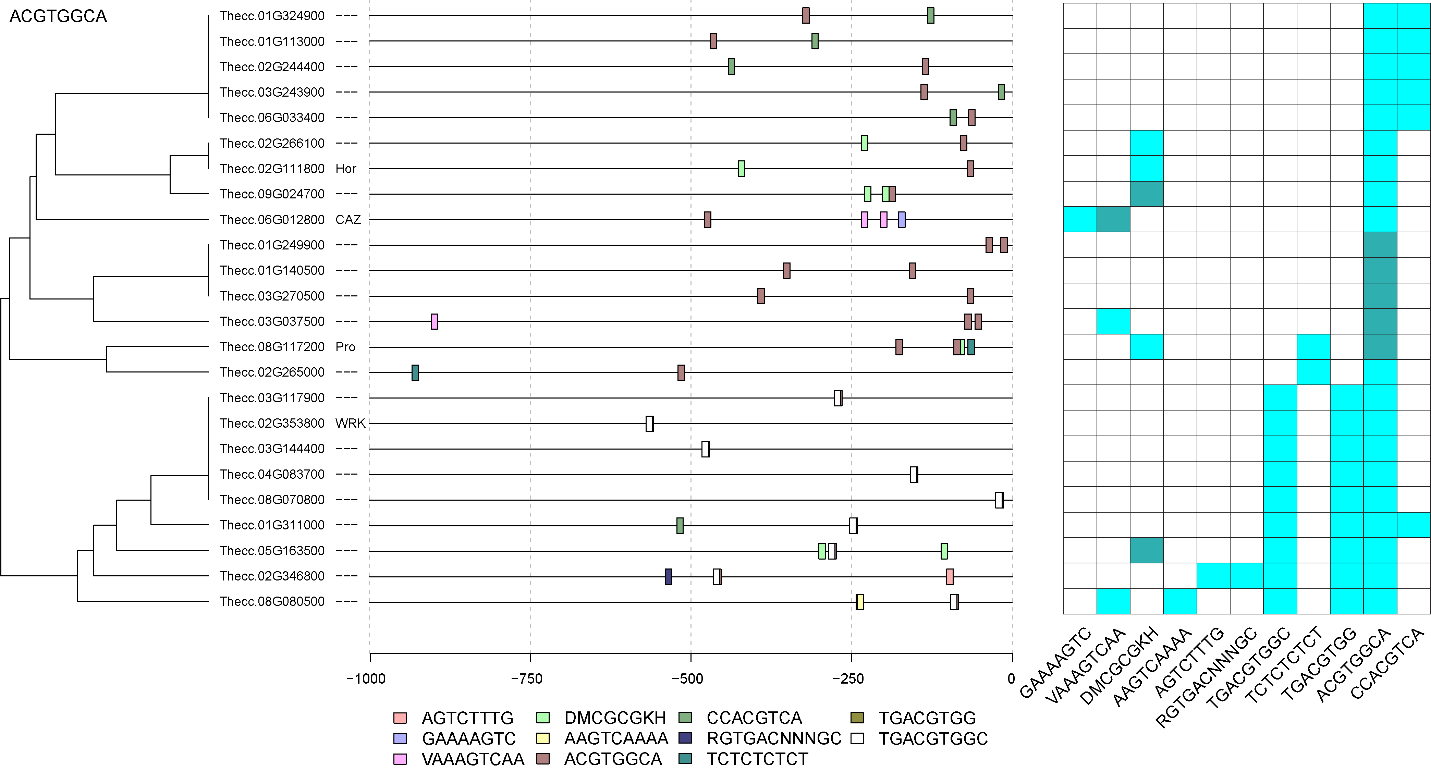


**Supplementary Figure S12. Upstream region diagrams of Group 6 genes with the LRENPCABE motif (ACGTGGCA).** Upstream sequences are right-aligned to correspond to the predicted TSS. Heat maps indicate the frequency of all motifs of interest per upstream sequence (white = 0, cyan = 1, teal = 2).


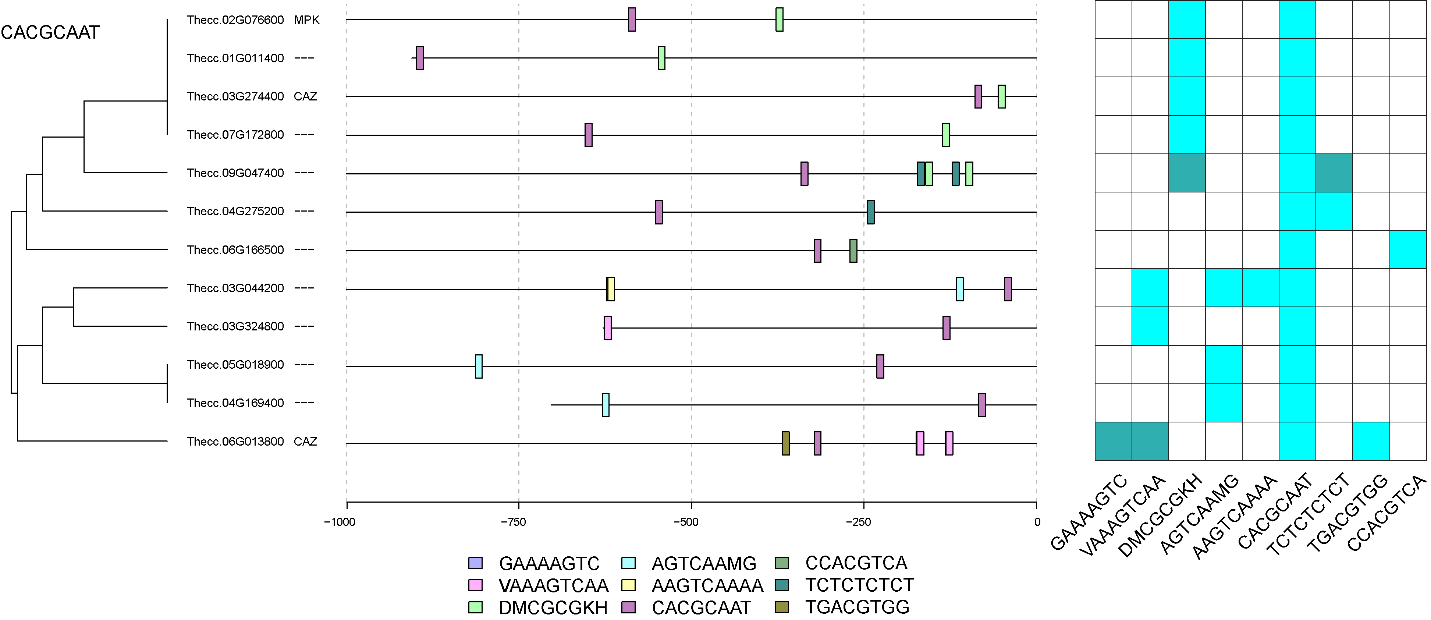


**Supplementary Figure S13. Upstream region diagrams of Group 6 genes with the CACGCAATGMGH3 motif (CACGCAAT).** Upstream sequences are right-aligned to correspond to the predicted TSS. Heat maps indicate the frequency of all motifs of interest per upstream sequence (white = 0, cyan = 1, teal = 2).


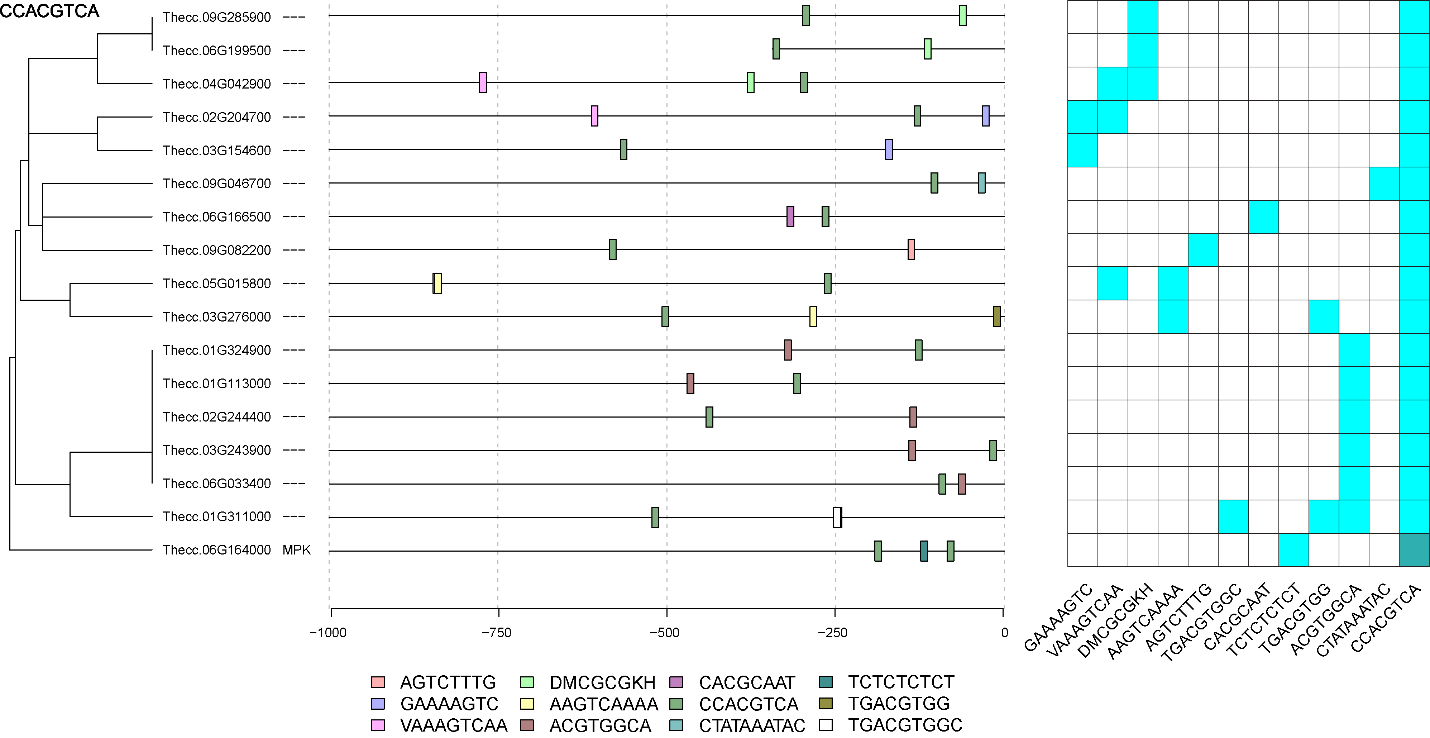


**Supplementary Figure S14. Upstream region diagrams of Group 6 genes with the UPRMOTIFIAT motif (CCACGTCA).** Upstream sequences are right-aligned to correspond to the predicted TSS. Heat maps indicate the frequency of all motifs of interest per upstream sequence (white = 0, cyan = 1, teal = 2).


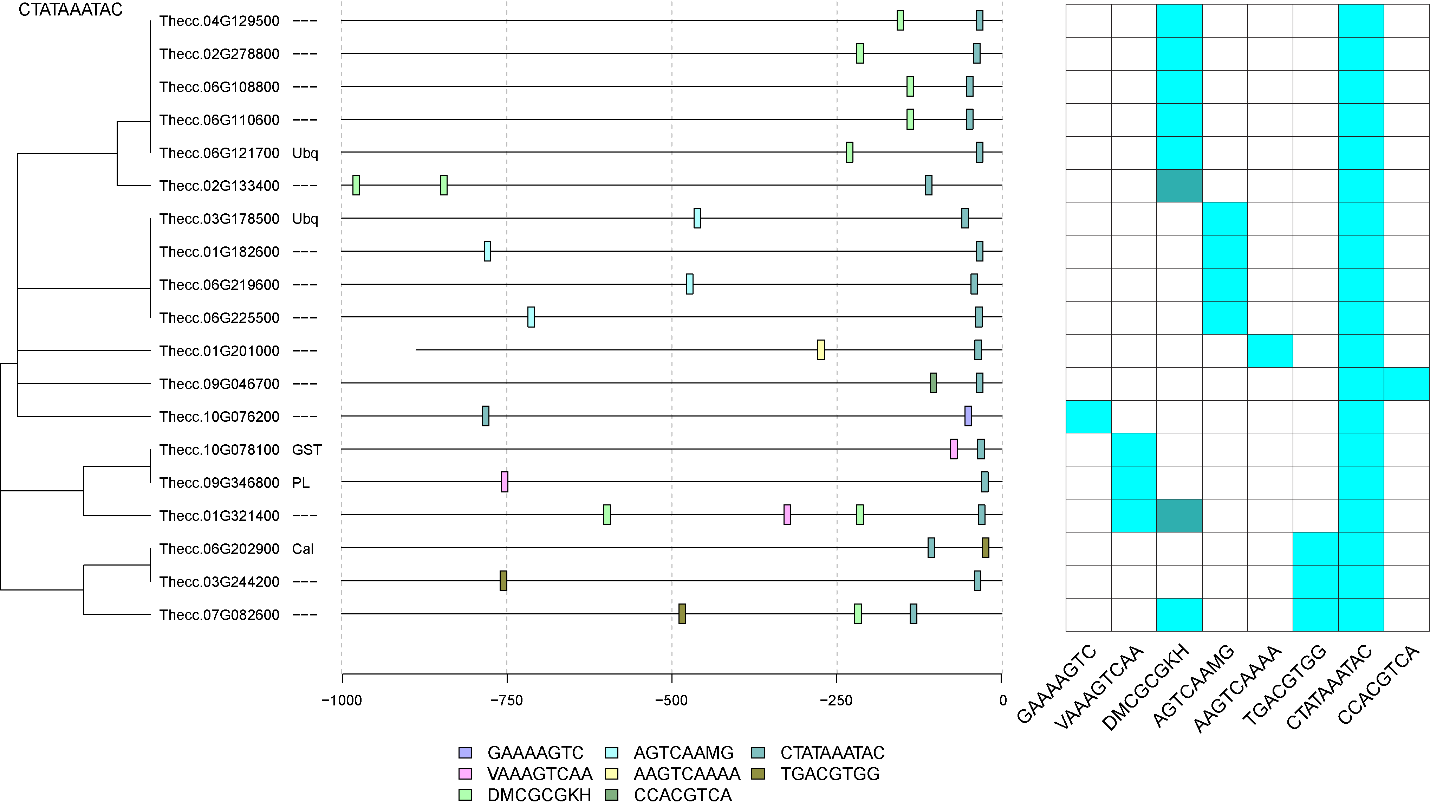


**Supplementary Figure S15. Upstream region diagrams of Group 6 genes with the TATABOX1 motif (CTATAAATAC).** Upstream sequences are right-aligned to correspond to the predicted TSS. Heat maps indicate the frequency of all motifs of interest per upstream sequence (white = 0, cyan = 1, teal = 2).


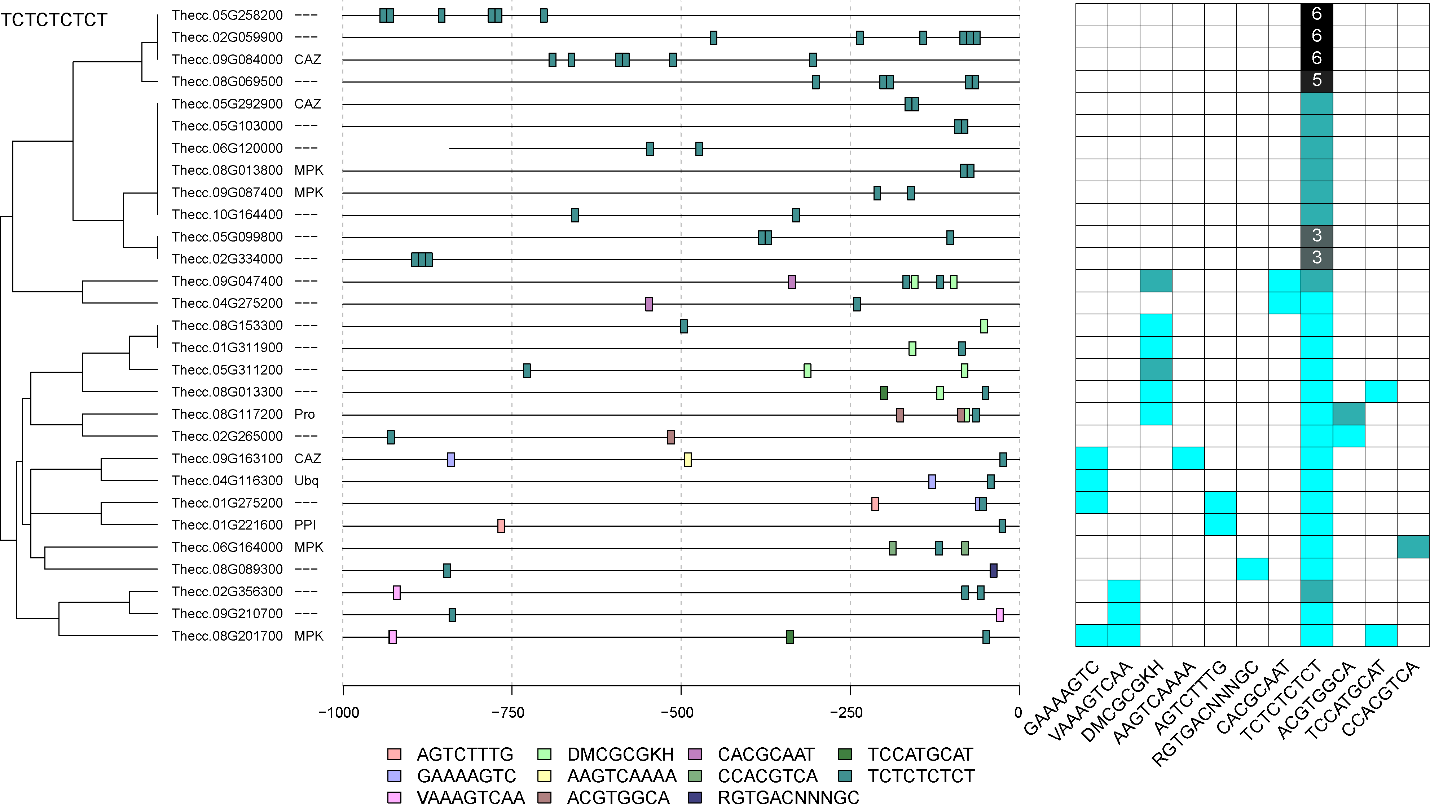


**Supplementary Figure S16. Upstream region diagrams of Group 6 genes with the CTRMCAMV35S motif (TCTCTCTCT).** Upstream sequences are right-aligned to correspond to the predicted TSS. Heat maps indicate the frequency of all motifs of interest per upstream sequence (white = 0, cyan = 1, teal = 2, frequencies >2 are indicated by the number on darker colors).


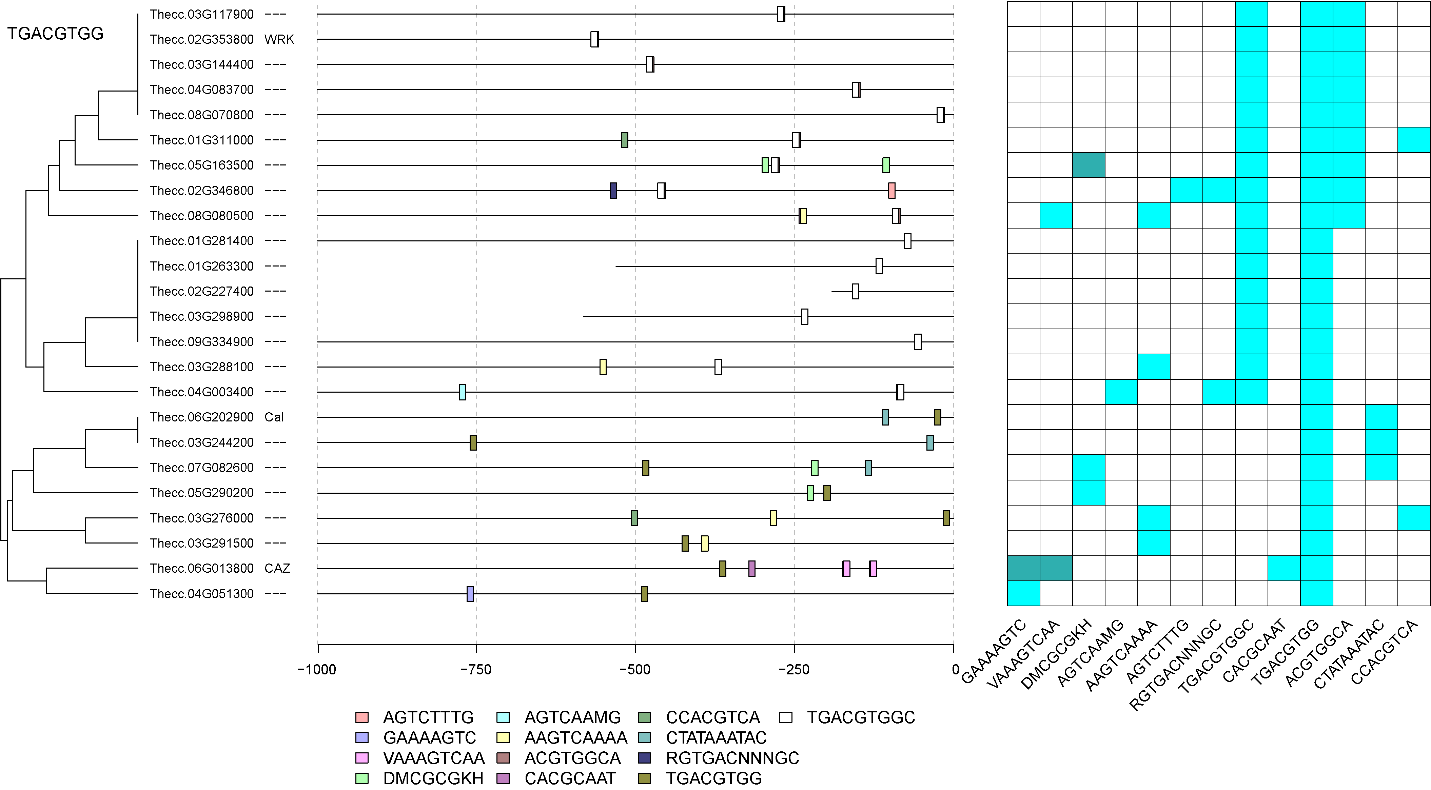


**Supplementary Figure S17. Upstream region diagrams of Group 6 genes with the HEXAT motif (TGACGTGG).** Upstream sequences are right-aligned to correspond to the predicted TSS. Heat maps indicate the frequency of all motifs of interest per upstream sequence (white = 0, cyan = 1, teal = 2).


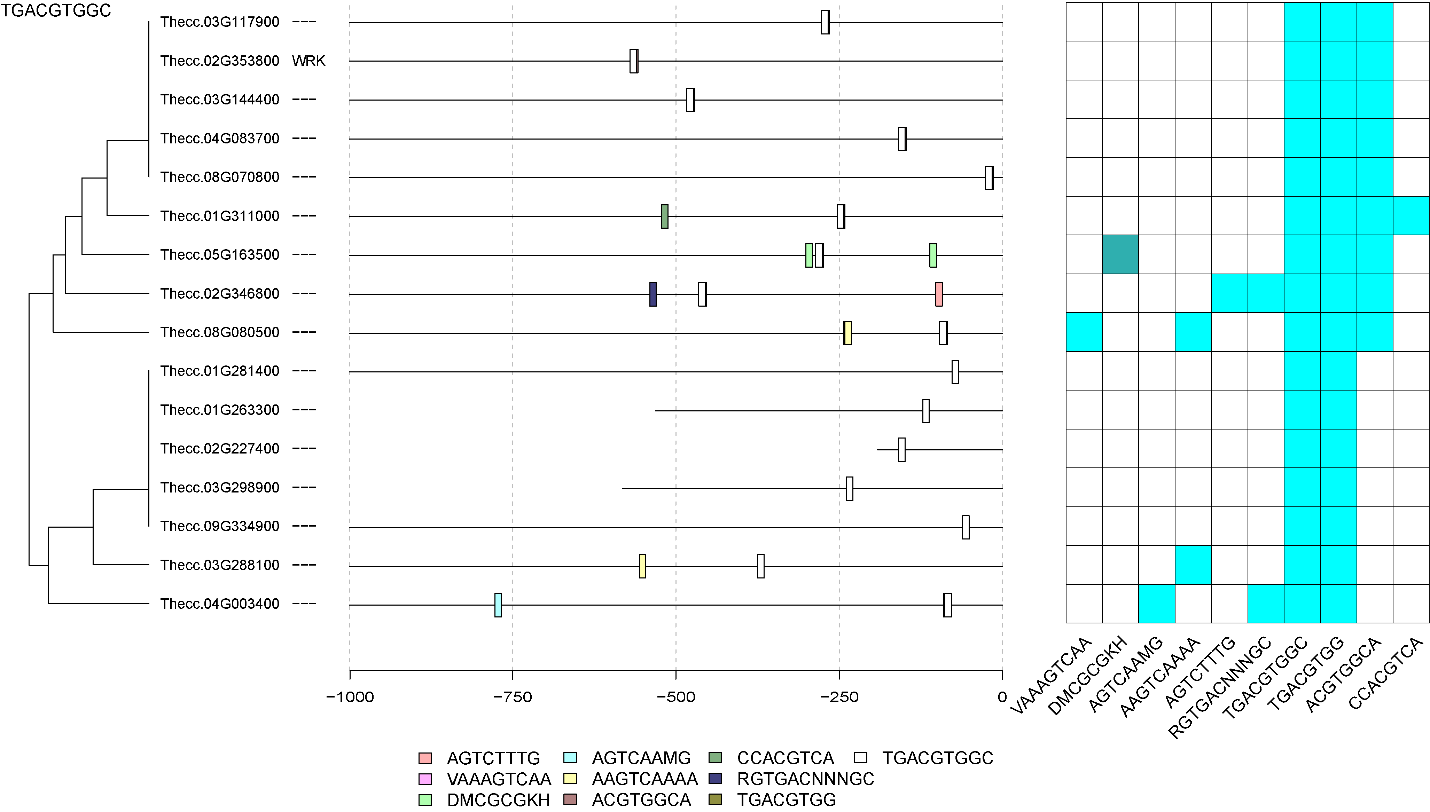


**Supplementary Figure S18. Upstream region diagrams of Group 6 genes with the AUXRETGA2GMGH3 motif (TGACGTGGC).** Upstream sequences are right-aligned to correspond to the predicted TSS. Heat maps indicate the frequency of all motifs of interest per upstream sequence (white = 0, cyan = 1, teal = 2).


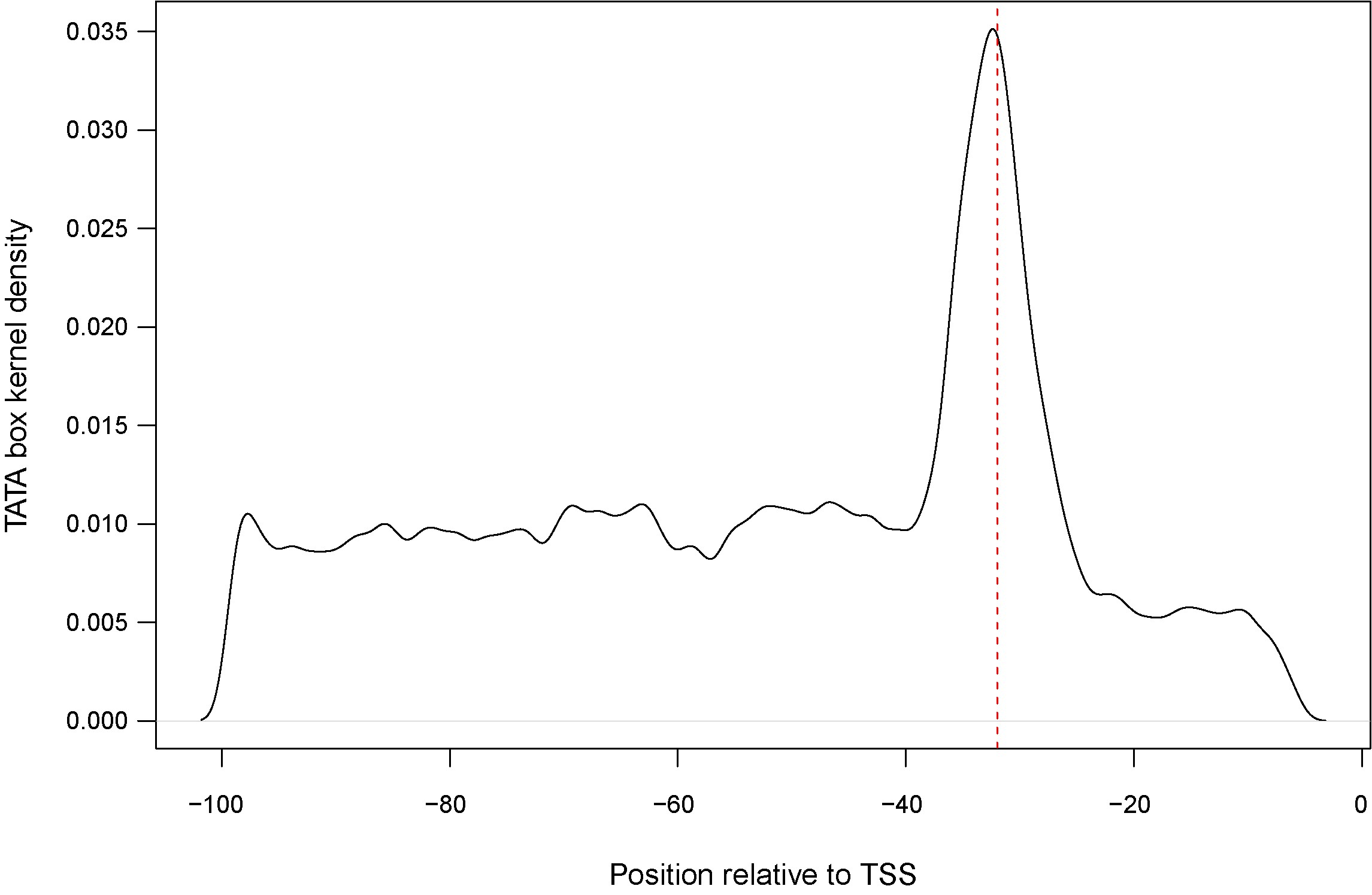


**Supplementary Figure S19. Kernel density of detected TATA box positions relative to the TSS.** TATA box and TATA-like motifs from PLACE DB were detected in regions upstream of cacao gene TSSs. The red line indicates the expected location of -32 bp upstream.

**Supplementary Table S1: Summary statistics of RNA-Seq data.** For each genotype and treatment, number of raw reads, adapter-trimmed reads, reads after purging sequences matching the *P. palmivora* genome, and reads mapped to Cacao genomic feature (i.e. protein-coding gene) are shown.

| **Cacao variety** | **Treatment** | **Replicate** | **# Raw Reads** | **# Trimmed Reads** | **# Post-Purge Reads** | **# Mapped to Feature** |
| --- | --- | --- | --- | --- | --- | --- |
| ICS1 | Control | 1 | 43,066,030 | 43,066,028 | 43,054,796 | 18,649,828 |
|  |  | 2 | 42,227,276 | 42,227,276 | 42,226,864 | 23,337,114 |
|  |  | 3 | 42,206,236 | 42,206,236 | 42,204,844 | 24,335,796 |
|  | *P. palmivora* | 1 | 42,946,882 | 42,946,878 | 38,199,098 | 21,697,162 |
|  |  | 2 | 43,276,208 | 43,276,202 | 42,317,174 | 22,789,484 |
|  |  | 3 | 42,303,724 | 42,303,724 | 40,354,058 | 18,139,912 |
| WFT | Control | 1 | 42,918,820 | 42,918,820 | 42,915,202 | 23,263,110 |
|  |  | 2 | 43,367,540 | 43,367,540 | 43,364,396 | 26,502,818 |
|  |  | 3 | 43,525,594 | 43,525,594 | 43,432,810 | 25,468,492 |
|  | *P. palmivora* | 1 | 44,217,852 | 44,217,850 | 37,080,202 | 19,052,610 |
|  |  | 2 | 42,377,080 | 42,377,080 | 29,807,574 | 14,022,860 |
|  |  | 3 | 40,746,342 | 40,746,342 | 36,393,472 | 19,653,576 |
| Gu133 | Control | 1 | 42,988,950 | 42,988,948 | 42,988,224 | 22,714,876 |
|  |  | 2 | 42,440,370 | 42,440,370 | 42,439,604 | 19,978,288 |
|  |  | 3 | 43,135,772 | 43,135,772 | 43,135,180 | 22,720,976 |
|  | *P. palmivora* | 1 | 42,858,394 | 42,858,394 | 33,420,224 | 18,340,834 |
|  |  | 2 | 42,255,650 | 42,255,648 | 39,766,752 | 22,060,464 |
|  |  | 3 | 42,458,234 | 42,458,234 | 33,572,568 | 17,003,206 |
| Spa9 | Control | 1 | 42,843,700 | 42,843,698 | 42,841,708 | 25,126,392 |
|  |  | 2 | 42,320,906 | 42,320,902 | 42,301,530 | 20,592,324 |
|  |  | 3 | 42,805,810 | 42,805,810 | 42,803,742 | 22,565,360 |
|  | *P. palmivora* | 1 | 42,993,518 | 42,993,518 | 38,659,784 | 19,484,674 |
|  |  | 2 | 43,310,734 | 43,310,732 | 38,549,236 | 22,344,850 |
|  |  | 3 | 42,275,236 | 42,275,236 | 41,251,688 | 21,416,552 |
| CCN51 | Control | 1 | 42,706,154 | 42,706,152 | 42,703,170 | 23,690,362 |
|  |  | 2 | 45,537,672 | 45,537,672 | 45,535,060 | 23,753,522 |
|  |  | 3 | 41,882,960 | 41,882,952 | 41,882,058 | 23,250,744 |
|  | *P. palmivora* | 1 | 43,944,840 | 43,944,840 | 34,769,944 | 21,195,358 |
|  |  | 2 | 43,078,362 | 43,078,360 | 32,850,186 | 18,613,884 |
|  |  | 3 | 42,604,084 | 42,604,084 | 36,035,158 | 19,079,748 |
| Sca6 | Control | 1 | 42,567,550 | 42,567,550 | 42,562,188 | 21,782,688 |
|  |  | 2 | 47,920,974 | 47,920,974 | 47,889,766 | 26,281,234 |
|  |  | 3 | 44,273,404 | 44,273,386 | 44,268,926 | 22,622,596 |
|  | *P. palmivora* | 1 | 42,815,162 | 42,815,162 | 39,121,278 | 22,656,524 |
|  |  | 2 | 42,742,928 | 42,742,928 | 35,523,938 | 16,140,074 |
|  |  | 3 | 43,019,776 | 43,019,772 | 26,213,266 | 14,286,296 |
| Pound7 | Control | 1 | 42,243,956 | 42,243,956 | 42,233,432 | 21,921,934 |
|  |  | 2 | 43,094,922 | 43,094,920 | 43,092,532 | 20,648,136 |
|  |  | 3 | 42,590,062 | 42,590,062 | 42,566,976 | 22,824,786 |
|  | *P. palmivora* | 1 | 43,359,728 | 43,359,728 | 37,898,316 | 21,106,696 |
|  |  | 2 | 43,390,312 | 43,390,312 | 33,336,842 | 18,021,074 |
|  |  | 3 | 42,748,070 | 42,748,070 | 41,037,810 | 22,283,492 |

**Supplementary Table S2. Numbers of differentially regulated KEGG family genes.** For each KEGG family, the numbers of genes up- or down-regulated per genotype shown.

| **Family** | **Total** | **ICS1** | **WFT** | **Gu133** | **Spa9** | **CCN51** | **Sca6** | **Pound7** |
| --- | --- | --- | --- | --- | --- | --- | --- | --- |
| **LRR** | 597 | 26 / 7 | 67 / 67 | 52 / 72 | 70 / 42 | 94 / 79 | 103 / 52 | 77 / 50 |
| **PR10** | 31 | 11 / 0 | 12 / 0 | 13 / 1 | 14 / 1 | 15 / 0 | 13 / 1 | 15 / 1 |
| **GLP** | 36 | 17 / 0 | 15 / 1 | 13 / 0 | 16 / 0 | 13 / 0 | 14 / 2 | 15 / 0 |
| **CYP** | 285 | 23 / 3 | 44 / 21 | 34 / 27 | 41 / 14 | 42 / 40 | 36 / 24 | 39 / 39 |
| **WRKY** | 59 | 19 / 0 | 31 / 3 | 23 / 4 | 30 / 5 | 32 / 8 | 30 / 8 | 29 / 10 |
| **ERF** | 104 | 11 / 0 | 40 / 6 | 17 / 4 | 33 / 3 | 37 / 8 | 26 / 6 | 35 / 7 |
| **MYB** | 187 | 9 / 1 | 23 / 11 | 16 / 11 | 22 / 4 | 25 / 20 | 18 / 22 | 18 / 19 |
| **GST** | 96 | 14 / 0 | 30 / 6 | 16 / 7 | 23 / 3 | 29 / 12 | 19 / 14 | 22 / 13 |
| **MAPK** | 47 | 9 / 0 | 17 / 6 | 11 / 4 | 15 / 5 | 17 / 9 | 16 / 4 | 14 / 8 |
| **Protease** | 324 | 17 / 7 | 28 / 57 | 25 / 28 | 27 / 35 | 21 / 68 | 30 / 47 | 32 / 48 |
| **UBQ** | 700 | 44 / 7 | 130 / 95 | 76 / 85 | 100 / 72 | 110 / 125 | 109 / 104 | 115 / 108 |
| **Total** | 2466 | 200 / 25 | 437 / 273 | 296 / 243 | 391 / 184 | 435 / 369 | 414 / 284 | 411 / 303 |

**Supplementary Table S3. All *de novo*-discovered motifs discovered from Cacao sequence upstream of the TSS.** All motifs, discovered from the differentially expressed genes sets shown, were tested for overrepresentation in correlation groups via Fisher’s exact test, and for associations with GO terms with GOMo.

| **Motif** | **Discovery** | **Correlation Group*** | **GO Term Association**** |
| --- | --- | --- | --- |
| GACTTTGTCAA | ICS1 Up  WFT Up | 6 | BP: defense response  MF: kinase activity |
| AGTCAARGAM | ICS1 Up | (1), 6 | - |
| CTTCTAGAA | ICS1 Up | 6 | BP: response to heat |
| CGCCGCCW | ICS1 Up | - | BP: DNA replication initiation  BP: embryonic development ending in seed dormancy  (BP: nitrogen compound metabolic process)  BP: transcription  BP: translation  (CC: chloroplast)  CC: chloroplast envelope  CC: chloroplast stroma  CC: CUL4 RING ubiquitin ligase complex  CC: cytosolic large ribosomal subunit  (CC: cytosolic ribosome)  (CC: large ribosomal subunit)  CC: mitochondrion  CC: nucleolus  (CC: ribosome)  MF: ATP-dependent helicase activity  MF: DNA-directed RNA polymerase activity  MF: nucleotide binding  MF: RNA binding  MF: structural constituent of ribosome  MF: translation initiation factor activity |
| TGGTCAAAD | ICS1 Up | 6 | BP: defense response  MF: kinase activity |
| GAAAAGTCAAAA | ICS1 Up | 6 | MF: carbohydrate binding  MF: kinase activity |
| AATTAACTGG | ICS1 Up | 6 | - |
| AACACGTGTT | ICS1 Up | 6 | - |
| CAAGGAAA | ICS1 Up | 6 | BP: transmembrane receptor protein tyrosine kinase signaling pathway  MF: protein serine/threonine kinase activity |
| GAAAACCAATA | ICS1 Down | - | - |
| GGTCAAAN | WFT Up | 6 | MF: kinase activity |
| GAAAAGTC | WFT Up  CCN51 Up | 6 | MF: kinase activity |
| ACACGYTW | WFT Up | (1), 6 | BP: response to water deprivation  BP: response to wounding |
| VAAAGTCAA | WFT Up | 6 | BP: defense response  CC: endomembrane system  MF: kinase activity |
| ADMCGCGKHT | WFT Up | 6, 10, 23 | CC: chloroplast  CC: mitochondrion |
| AGCCGCCA | WFT Up  Sca6 Up | 6 | BP: biotin biosynthetic process  (BP: metabolic process)  BP: transcription  BP: translation  (CC: chloroplast)  CC: chloroplast stroma  CC: CUL4 RING ubiquitin ligase complex  CC: cytosolic large ribosomal subunit  (CC: cytosolic ribosome)  (CC: mitochondrion)  CC: mitochondrial inner membrane  CC: nucleolus  (CC: ribosome)  (MF: binding)  MF: DNA-directed RNA polymerase activity  MF: helicase activity  MF: nucleotide binding  MF: RNA binding  MF: structural constituent of ribosome |
| AAAATAATACT | WFT Up | 6 | (BP: regulation of transcription)  BP: regulation of transcription, DNA-dependent  CC: endomembrane system  (MF: DNA binding)  MF: transcription factor activity |
| ACGAAGGC | WFT Up | - | CC: chloroplast  CC: mitochondrion  MF: ATP-dependent helicase activity |
| CGTCAAAG | WFT Up | 6 | - |
| ACCGAGTCA | WFT Up | - | CC: chloroplast  CC: mitochondrion |
| ACGTGGAA | WFT Up | - | BP: response to wounding  CC: chloroplast  CC: endoplasmic reticulum |
| AAATATYTTTTAAAA | WFT Down | - | CC: endomembrane system  (MF: DNA binding)  MF: hydrolase activity, hydrolyzing O-glycosyl compounds  MF: transcription factor activity |
| CCAACGGCTAS | WFT Down | - | - |
| CCGACAAGAGCC | WFT Down | - | (CC: chloroplast)  CC: chloroplast envelope  CC: chloroplast thylakoid membrane  CC: mitochondrion |
| AAAATTTATTTGAY | WFT Down | - | CC: endomembrane system  MF: transcription factor activity |
| GACTTGGAA | Gu133 Up | - | BP: defense response |
| DMCGCGKH | Gu133 Up  Pound7 Up | (1), 6, 10, 23 | CC: chloroplast  CC: mitochondrion |
| ACACGCTTKCK | Gu133 Up | - | - |
| GGGTATWTATAGRNR | Gu133 Up | - | BP: carbohydrate metabolic process  BP: lipid transport  BP: plant-type cell wall loosening  BP: response to auxin stimulus  BP: response to gibberellin stimulus  BP: response to wounding  CC: endomembrane system  CC: extracellular region  (MF: hydrolase activity, hydrolyzing O-glycosyl compounds)  MF: lipid binding  MF: polygalacturonase activity  MF: transcription factor activity |
| TCAAGGAACTA | Gu133 Up | 6 | - |
| AGAATTTTCCAAGG | Gu133 Up | - | BP: defense response |
| TGGTCAAAAA | Gu133 Up | - | MF: kinase activity |
| CAAGGAAAGT | Gu133 Up | - | MF: transcription factor activity |
| CTTCTAGA | Gu133 Up  Pound7 Up | 6 | - |
| AGAAAAGTCTM | Gu133 Up | 6 | CC: mitochondrion |
| MGCCGCCA | Gu133 Up | 6 | BP: transcription  BP: translation  (CC: chloroplast)  CC: chloroplast envelope  CC: chloroplast stroma  CC: chloroplast thylakoid membrane  CC: CUL4 RING ubiquitin ligase complex  CC: cytosolic large ribosomal subunit  (CC: cytosolic ribosome)  (CC: large ribosomal subunit)  CC: mitochondrial inner membrane  (CC: mitochondrion)  CC: nucleolus  (CC: ribosome)  MF: ATP-dependent helicase activity  (MF: binding)  MF: DNA-directed RNA polymerase activity  (MF: helicase activity)  MF: nucleotide binding  MF: oxidoreductase activity, acting on the aldehyde or oxo group of donors, NAD or NADP as acceptor  MF: protein transporter activity  MF: RNA binding  MF: structural constituent of ribosome  MF: translation initiation factor activity |
| CTCTCAAC | Gu133 Down | - | (CC: chloroplast)  CC: chloroplast thylakoid membrane  CC: mitochondrial matrix |
| AASCGCGTKGRV | Spa9 Up | 6 | CC: chloroplast  CC: mitochondrion |
| CGCCGCCN | Spa9 Up | 6 | BP: mitochondrial transport  BP: transcription  BP: translation  (CC: chloroplast)  CC: chloroplast envelope  CC: chloroplast stroma  CC: CUL4 RING ubiquitin ligase complex  CC: cytosolic large ribosomal subunit  (CC: cytosolic ribosome)  (CC: large ribosomal subunit)  CC: mitochondrial inner membrane  (CC: mitochondrion)  CC: nucleolus  (CC: ribosome)  MF: ATP-dependent helicase activity  (MF: binding)  MF: DNA-directed RNA polymerase activity  (MF: helicase activity)  MF: nucleotide binding  MF: RNA binding  MF: structural constituent of ribosome  MF: translation initiation factor activity |
| CTTTGACGTTTCC | Spa9 Up | - | - |
| CTATAAATACCCM | Spa9 Up | 6 | BP: carbohydrate metabolic process  BP: cellular response to phosphate starvation  BP: lipid transport  BP: multicellular organismal development  BP: oligopeptide transport  BP: plant-type cell wall loosening  BP: plant-type cell wall modification during multidimensional cell growth  BP: response to auxin stimulus  CC: endomembrane system  CC: extracellular region  (MF: hydrolase activity, hydrolyzing O-glycosyl compounds)  MF: lipid binding  MF: pectinesterase inhibitor activity  MF: peroxidase activity  MF: polygalacturonase activity  MF: transcription factor activity  MF: water channel activity |
| AGTTTTTGTCTCM | Spa9 Up | 6 | - |
| TTGGTCAAAMHVR | Spa9 Up | 6 | BP: response to chitin |
| WGGTCAAA | Spa9 Up | 6 | MF: kinase activity |
| ACMAAGTCAAVGWC | Spa9 Up | - | BP: defense response  MF: kinase activity |
| ACGCGGYK | CCN51 Up | 6, 10 | BP: mitochondrial transport  BP: translation  CC: chloroplast  CC: mitochondrion  MF: nucleotide binding  MF: structural constituent of ribosome |
| AGTCAAMG | CCN51 Up | (1), 6 | BP: defense response  MF: kinase activity |
| CACGGCTT | CCN51 Up | - | CC: mitochondrion |
| TGGTCAAMMD | CCN51 Up | 6 | MF: kinase activity |
| ACGTGTGCGD | CCN51 Up | - | CC: chloroplast  CC: mitochondrion  MF: chlorophyll binding  MF: nutrient reservoir activity |
| TCAAAGCAAAAATT | CCN51 Down | - | CC: endomembrane system |
| TACACTTAAT | CCN51 Down | - | BP: regulation of transcription  CC: endomembrane system  MF: transcription factor activity |
| AAGTCAAAA | Sca6 Up | 6 | BP: defense response  BP: response to chitin  CC: endomembrane system  MF: kinase activity |
| TCTAGAAGG | Sca6 Up | 6 | BP: response to heat  BP: translation  CC: mitochondrion |
| AACGCGTAAAA | Sca6 Up | - | - |
| AGTCTTTG | Sca6 Up | 6 | BP: defense response |
| TGGTCAAA | Sca6 Up | 6 | - |
| AACGKCAT | Sca6 Up | - | - |
| ACGTCAAA | Sca6 Up | 6 | - |
| AACGTGTGCG | Sca6 Up | - | BP: regulation of transcription, DNA-dependent  MF: transcription factor activity |
| ACCGACCD | Sca6 Up | 6 | CC: chloroplast  CC: mitochondrion |
| AGACTTTGTCAAGWA | Sca6 Up | - | - |
| ACGCGGYTKSV | Sca6 Up | - | CC: chloroplast  CC: mitochondrion |
| GAARYTTCCACG | Sca6 Up | 6 | BP: response to heat  CC: chloroplast |
| ATCATCAATTA | Sca6 Down | - | CC: endomembrane system |
| GTCATTTTCA | Sca6 Down | - | - |
| AAGAGAGAG | Sca6 Down | - | BP: chlorophyll biosynthetic process  (BP: flower development)  BP: fruit development  (BP: leaf development)  BP: leaf morphogenesis  BP: meristem initiation  BP: mitochondria-nucleus signaling pathway  (BP: multicellular organismal development)  BP: ovule development  BP: polarity specification of adaxial/abaxial axis  BP: positive regulation of transcription  BP: potassium ion transport  BP: protein amino acid phosphorylation  (BP: regulation of transcription)  BP: regulation of transcription, DNA-dependent  BP: response to brassinosteroid stimulus  BP: transmembrane receptor protein tyrosine kinase signaling pathway  BP: xylem and phloem pattern formation  CC: cytoplasm  CC: nucleus  CC: plasma membrane  MF: ATP binding  (MF: DNA binding)  (MF: kinase activity)  MF: microtubule motor activity  MF: potassium ion transmembrane transporter activity  MF: protein binding  MF: protein serine/threonine kinase activity  MF: transcription activator activity  MF: transcription factor activity |
| CAATAATTAGTAD | Sca6 Down | - | CC: endomembrane system |
| TGGTCAAMVT | Pound7 Up | 6 | - |
| CTTTGACTW | Pound7 Up | 6 | BP: defense response |
| ACCGAGGAA | Pound7 Up | - | BP: metal ion transport  CC: chloroplast  CC: mitochondrion  MF: metal ion transmembrane transporter activity |
| MGCCGCCR | Pound7 Up | 6 | BP: embryonic development ending in seed  BP: transcription  BP: translation  (CC: chloroplast)  CC: chloroplast envelope  CC: chloroplast stroma  CC: CUL4 RING ubiquitin ligase complex  CC: cytosolic large ribosomal subunit  (CC: cytosolic ribosome)  (CC: large ribosomal subunit)  CC: mitochondrial inner membrane  (CC: mitochondrion)  CC: nucleolus  (CC: ribosome)  MF: ATP-dependent helicase activity  (MF: binding)  MF: DNA-directed RNA polymerase activity  (MF: helicase activity)  MF: nucleotide binding  MF: RNA binding  MF: structural constituent of ribosome  MF: translation initiation factor activity |
| AVMGTCVAAS | Pound7 Up | 6 | - |
| AACACGCTTTA | Pound7 Up | - | - |
| CTATAAATACCM | Pound7 Up | 6 | BP: carbohydrate metabolic process  BP: lipid transport  BP: plant-type cell wall loosening  BP: plant-type cell wall modification during multidimensional cell growth  BP: response to auxin stimulus  CC: endomembrane system  MF: lipid binding  MF: polygalacturonase activity  MF: transcription factor activity |
| ACGTGGCRWGWACYS | Pound7 Up | - | BP: photosynthesis  BP: response to abscisic acid stimulus  BP: response to cold  BP: response to desiccation  BP: response to heat  (BP: response to stress)  (BP: response to water deprivation)  BP: vegetative to reproductive phase transition of meristem  (CC: chloroplast)  CC: chloroplast stroma  CC: chloroplast thylakoid lumen  CC: chloroplast thylakoid membrane  CC: endoplasmic reticulum  CC: plastoglobule  CC: thylakoid  MF: chlorophyll binding |

*motifs are considered over-represented or under-represented (parentheses) in correlation groups following two-sided Fisher’s exact test (p ≤ 0.05); **BP = biological process, CC = cellular component, MF = molecular function

**Supplementary Table S4. Potential *cis-*regulatory modules and their relative locations and count in upstream regions of correlation group 6 genes.** Putative motif associations were discovered by manually inspecting the motif layout for each motif of interest (from Tables 2 and 3).

| **Module** | **Upstream Motif** | **Downstream Motif** | **Notes** | **Count** |
| --- | --- | --- | --- | --- |
| CRM1 | DMCGCGKH | AGTCTTTG | Distance < 100 bp | 3 |
| CRM2 | AGTCAAMG | AGTCTTTG | Distance < 250 bp | 3 |
| CRM3 | GAAAAGTC | DMCGCGKH | Distance < 100 bp | 5 |
| CRM4 | VAAAGTCAA | VAAAGTCAA | Distance < 100 bp | 6 |
| CRM5 | VAAAGTCAA | DMCGCGKH | Distance < 100 bp | 5 |
| CRM6 | DMCGCGKH | DMCGCGKH | Distance < 250 bp | 18 |
| CRM7 | DMCGCGKH | TCTCTCTCT | Distance < 100 bp | 5 |
| CRM8 | DMCGCGKH | CTATAAATAC | Distance < 250 bp | 5 |
| CRM9 | RGTGACNNNGC | DMCGCGKH | Distance < 100 bp | 3 |
| CRM10 | AGTCAAMG | DMCGCGKH | Distance < 250 bp | 6 |
| CRM11 | DMCGCGKH | ATGCAAMG | Distance < 250 bp | 4 |
| CRM12 | AGTCAAMG | CTATAAATAC | Distance > 250 bp | 4 |
| CRM13 | ACGTGGCA | CCACGTCA | Distance < 250 bp | 3 |
| CRM14 | ACGTGGCA | ACGTGGCA | Distance < 250 bp | 4 |
| CRM15 | DMCGCGKH | ACGTGGCA | Distance < 250 bp | 3 |
| CRM16 | CACGCAAT | DMCGCGKH | Distance < 250 bp | 4 |
| CRM17 | TCTCTCTCT | TCTCTCTCT | Distance < 5 bp,  or Distance < 100 bp | 21 |

**Supplementary Table S5. Selected genes from correlation Group 6 with putative CRMs in the upstream regions.** All loci, annotations, KEGG family (if present), and putative CRMs are shown for genes from Group 6 with one or more putative CRMs.

| **Locus** | **Annotation** | **KEGG Family** | **CRMs** |
| --- | --- | --- | --- |
| Thecc.08G052700 | Pyridoxal-dependent decarboxylase family protein | --- | 1 |
| Thecc.04G125500 | (1 of 15) 2.1.1.50 - Loganate O-methyltransferase | --- | 2 |
| Thecc.01G415400 | O-fucosyltransferase family protein (71%T) | CAZ | 3 |
| Thecc.08G090400 | Salt tolerance zinc finger | --- | 3 |
| Thecc.01G264300 | Inhibitor of trypsin and hageman factor (79%P) | Prot | 4 |
| Thecc.06G012400 | FAD-binding and BBE domain-containing protein | CAZ | 4 |
| Thecc.06G012800 | (1 of 34) 1.3.3.8 - Tetrahydroberberine oxidase / THB oxidase | CAZ | 4 |
| Thecc.06G013800 | (1 of 34) 1.3.3.8 - Tetrahydroberberine oxidase / THB oxidase | CAZ | 4 |
| Thecc.07G069300 | Diacylglycerol kinase 5 (63%T) | --- | 4 |
| Thecc.09G110900 | Serine/threonine-protein kinase PBS1 | Lec | 4 |
| Thecc.07G127000 | SecY protein transport family protein (91%T) | --- | 5 |
| Thecc.08G038200 | Alpha/beta-Hydrolases superfamily protein (75%T) | --- | 5 |
| Thecc.02G133400 | NA | --- | 6 |
| Thecc.02G174100 | Secretory carrier membrane protein (SCAMP) family protein (75%T) | --- | 6 |
| Thecc.02G189500 | ARM repeat superfamily protein (66%P) | Ubq | 6 |
| Thecc.02G306400 | ARM repeat superfamily protein | Ubq | 6 |
| Thecc.03G315200 | Flavonol-3-O-glycoside-7-O-glucosyltransferase 1 | CAZ | 6 |
| Thecc.04G065900 | Galacturonosyltransferase-like 10 (69%T) | CAZ | 6 |
| Thecc.05G119700 | calmodulin-binding protein of 25 kDa | Clm | 6 |
| Thecc.05G171100 | (1 of 25) PF05678 - VQ motif (VQ) | --- | 6 |
| Thecc.05G311200 | Soybean gene regulated by cold-2 | --- | 6 |
| Thecc.06G164800 | ER lumen protein retaining receptor family protein (87%T) | --- | 6 |
| Thecc.09G148400 | HCO3- transporter family (72%T) | --- | 6 |
| Thecc.09G251500 | Cystatin B (67%T) | --- | 6 |
| Thecc.01G311900 | uncharacterized protein (47%P) | --- | 7 |
| Thecc.08G013300 | RNA-binding protein 47C (73%P) | --- | 7 |
| Thecc.01G321400 | Phosphatase 2C 49 (74%P) | --- | 8 |
| Thecc.04G129500 | Class I glutamine amidotransferase-like superfamily protein (78%T) | --- | 8 |
| Thecc.06G108800 | Inorganic pyrophosphatase 2 (93%P) | --- | 8 |
| Thecc.06G110600 | Inorganic pyrophosphatase 2 (93%P) | --- | 8 |
| Thecc.06G121700 | Cytochrome p450 superfamily protein (76%P) | CYP | 8 |
| Thecc.02G251000 | uncharacterized protein (48%P) | --- | 9 |
| Thecc.04G114000 | Myb domain protein 20 (65%P) | Myb | 9 |
| Thecc.05G201900 | Mitogen-activated protein kinase kinase kinase 19 | MAPK | 9 |
| Thecc.02G065400 | Disease resistance-responsive family protein (72%P) | --- | 10 |
| Thecc.02G291800 | Nudix hydrolase (71%T) | --- | 10 |
| Thecc.10G027600 | Nucleotide-sugar transporter family protein (94%T) | --- | 10 |
| Thecc.04G044400 | GRAM domain family protein (70%P) | --- | 11 |
| Thecc.04G169100 | Carrot EP3-3 chitinase (75%T) | CAZ | 11 |
| Thecc.09G258400 | Seven transmembrane MLO family protein (76%P) | --- | 11 |
| Thecc.01G182600 | Ethylene-forming enzyme (78%T) | --- | 12 |
| Thecc.03G178500 | Cytochrome p450 | CYP | 12 |
| Thecc.06G219600 | uncharacterized protein (42%P) | --- | 12 |
| Thecc.06G225500 | Stigma-specific Stig1 family protein | --- | 12 |
| Thecc.01G113000 | Shikimate kinase | --- | 13 |
| Thecc.01G324900 | uncharacterized protein (15%T) | --- | 13 |
| Thecc.03G243900 | Ras 5 (92%T) | --- | 13 |
| Thecc.01G140500 | NAC domain transcriptional regulator superfamily protein (71%P) | --- | 14 |
| Thecc.01G249900 | (1 of 2) 3.5.99.2 - Aminopyrimidine aminohydrolase / Thiaminase II | --- | 14 |
| Thecc.03G037500 | Zinc finger protein-related (66%T) | --- | 14 |
| Thecc.02G111800 | Auxin-responsive GH3 family protein (83%T) | Hor | 15 |
| Thecc.02G266100 | Transducin/WD40 repeat-like superfamily protein (63%P) | --- | 15 |
| Thecc.02G076600 | GATA transcription factor 5 | TF | 16 |
| Thecc.03G274400 | Osmotin 34 (63%T) | CAZ | 16 |
| Thecc.02G059900 | SBP family protein | Ubq | 17 |
| Thecc.02G334000 | C2H2-like zinc finger protein | --- | 17 |
| Thecc.02G356300 | uncharacterized protein (52%P) | --- | 17 |
| Thecc.05G099800 | NA | --- | 17 |
| Thecc.05G103000 | Hydroxyproline-rich glycoprotein family protein | --- | 17 |
| Thecc.05G258200 | uncharacterized protein (80%P) | --- | 17 |
| Thecc.05G292900 | O-Glycosyl hydrolases family 17 protein (76%T) | CAZ | 17 |
| Thecc.06G120000 | unknown | --- | 17 |
| Thecc.08G013800 | Heat stress transcription factor A-2c | TF | 17 |
| Thecc.08G069500 | AT-hook DNA-binding family protein (60%P) | --- | 17 |
| Thecc.09G084000 | UDP-Glycosyltransferase superfamily protein (66%P) | CAZ | 17 |
| Thecc.06G085800 | (1 of 4) K17302 - coatomer, subunit beta' (COPB2, SEC27) | --- | 1, 2, 5, 10 |
| Thecc.06G086100 | (1 of 4) K17302 - coatomer, subunit beta' (COPB2, SEC27) | --- | 1, 2, 5, 10 |
| Thecc.01G283300 | (1 of 2) 1.2.1.70 - Glutamyl-tRNA reductase | --- | 3, 5 |
| Thecc.02G116600 | uncharacterized protein (51%P) | --- | 6, 10, 11 |
| Thecc.09G024700 | uncharacterized protein (48%P) | --- | 6, 15 |
| Thecc.08G117200 | Mitogen-activated protein kinase kinase kinase 1 | MAPK | 7, 14 |
| Thecc.09G047400 | (1 of 3) 1.1.1.40 - Malate dehydrogenase (oxaloacetate-decarboxylating) (NADP(+)) / Pyruvic-malic carboxylase | --- | 7, 16 |
